# Human papillomavirus E7 binds Oct4 and regulates its activity in HPV-associated cervical cancers

**DOI:** 10.1371/journal.ppat.1008468

**Published:** 2020-04-16

**Authors:** Theofano Panayiotou, Stella Michael, Apostolos Zaravinos, Ece Demirag, Charis Achilleos, Katerina Strati

**Affiliations:** 1 Department of Biological Sciences, University of Cyprus, Nicosia, Cyprus; 2 College of Medicine, Member of QU Health, Qatar University, Doha, Qatar; 3 Department of Life Sciences, European University Cyprus, Nicosia, Cyprus; University of Wisconsin Madison School of Medicine and Public Health, UNITED STATES

## Abstract

Octamer binding transcription factor-4 (Oct4), is highly expressed in stem cells and has indispensable roles in pluripotency and cellular reprogramming. In contrast to other factors used for cellular reprogramming, a role for Oct4 outside embryonic stem cells has been elusive and highly controversial. Emerging evidence implicates Oct4 in the carcinogenic process, but the mechanism through which Oct4 may be functioning in cancers is not fully appreciated. Here, we provide evidence that Oct4 is expressed in human cervical cancer and this expression correlates with the presence of the human papillomavirus (HPV) oncogenes E6 and E7. Surprisingly, the viral oncogenes can complement exogenously provided Oct4 in reprogramming assays, providing functional validation for their ability to activate Oct4 transcription in Mouse Embryonic Fibroblasts (MEFs). To interrogate potential roles of Oct4 in cervical cancers we knocked-down Oct4 in HPV(+) (HeLa & CaSki) and HPV(-) (C33A) cervical cancer cell lines and found that Oct4 knockdown attenuated clonogenesis, only in the HPV(+) cells. More unexpectedly, cell proliferation and migration, were differentially affected in HPV(+) and HPV(-) cell lines. We provide evidence that Oct4 interacts with HPV E7 specifically at the CR3 region of the E7 protein and that introduction of the HPV oncogenes in C33A cells and human immortalised keratinocytes generates Oct4-associated transcriptional and phenotypic patterns, which mimic those seen in HPV(+) cells. We propose that a physical interaction of Oct4 with E7 regulates its activity in HPV(+) cervical cancers in a manner not seen in other cancer types.

## Author summary

The transcription factor Oct4 with well-known roles in embryogenesis, pluripotency and cellular reprogramming has recently been found to be expressed in several types of somatic tumors. Even though its role in cancer remains controversial, we provide evidence that Oct4 is expressed in cervical cancer tissues and cancer cell lines. The viral oncogenes of the Human Papillomavirus significantly elevate Oct4 expression both in normal and cancer cells, likely through transcriptional upregulation. While the expression levels of Oct4 in cancer are low compared to those seen in stem cells, our results suggest that they are still consequential to cell proliferation, self-renewal, and migration. We demonstrate a physical interaction of the E7 oncoprotein with Oct4, mapping to the CR3 region of E7, which correlates to a distinct Oct4 transcriptional output. Introduction of E7 into HPV(-) cells and immortalised human keratinocytes led to transcriptional and phenotypic changes, which mimicked results in HPV(+) cells. These insights provide a plausible mechanism and consequences for a long-suspected interaction.

## Introduction

Octamer-binding transcription factor-4 (Oct4) encodes a transcription factor that contains a POU homeodomain and has the ability to regulate genes involved in embryonic development, stem cell pluripotency, and self-renewal [1, 2]. It is also considered as a ‘master regulator’ that controls the de-differentiation process of somatic cells towards an induced pluripotent state [3]. Nevertheless, evidence for roles of Oct4 in tissue stem cells and somatic cells has been scarce and controversial [4, 5].

The clearest evidence for non-pluripotency-related functions of Oct4, lies in its involvement in some cancer types. Oct4 is broadly expressed in the lung and the small intestine, and its aberrant expression is associated with tumorigenesis in adult tissues, including testicular germ cell cancers [6, 7]. Initial evidence suggests that Oct4 acts as a rheostat gene controlling the development and progression of malignancy in germ cells [6]. More recent evidence reports the expression of Oct4 in somatic malignancies, such as prostate [8], breast [9], ovarian [10], hepatocellular [11], head and neck [12] and cervical [13] cancers. As a cancer marker, Oct4 was recently associated with poor prognosis in hepatocellular carcinomas [14]. However, its mechanistic role in cancer remains obscure and may be further complicated by the existence of different isoforms, resulting from alternative splicing, or usage of alternative AUG and non-AUG translation initiation codons [15]. All Oct4 isoforms have been implicated in cancer, including Oct4A, the isoform with the most established roles in pluripotency [16].

Nevertheless, Oct4 is not the only pluripotency-related gene to be linked to carcinogenesis. In fact, evidence is mounting for a role of Sox2, Nanog and others in epithelial malignancies. For example, Nanog has been shown to regulate proliferation in a lineage-restricted fashion in stratified epithelia and contribute to carcinogenesis [17]. In light of the fact that two of the factors used in the original reprogramming cocktail (c-Myc and Klf4) had established roles in carcinogenesis, it is crucial to understand the degree to which the roles of these proteins overlap in pluripotency and cancer.

Malignancy in the cervical epithelium is associated with persistent human papilloma virus (HPV) infection. Enhanced plasticity in the cervical epithelium is also noted after HPV infection, suggesting that the viral oncogenes can induce changes in a number of genes involved in tissue reorganisation. The two viral oncogene products (E6 and E7) being expressed in HPV-associated cervical cancers, target p53 and pRb, respectively. The attenuation of the p53 and pRb pathways, in addition to contributing to carcinogenesis, has been implicated in the increase of cellular plasticity. The pRb protein has been shown to bind and inhibit the promoters of Oct4 and Sox2 [18], whereas p53 has been shown to negatively regulate Nanog in embryonic stem cells [19]. Both pathways have been shown to be critical barriers during reprogramming to pluripotency [3, 20, 21]. Despite these lines of evidence which hint at overlap between the activity of the E6 and E7 oncogene products, tissue plasticity and cancer, it is not known whether they directly regulate Oct4 in the context of cancer.

In this study, we investigated the role of Oct4 in HPV(+) and HPV(-) cervical cancer samples and cell lines, and further examined whether the E6 and E7 oncogenes can affect the expression and properties of Oct4 in HPV-associated cervical tumors. Our data show an elevated expression of Oct4 in HPV(+) cancer samples and cell lines, compared to the HPV(-) ones, and we provide a link between the viral oncogenes and the elevated Oct4 levels in these cells. In addition, we provide evidence for a physical interaction between the CR3 domain of E7 and Oct4, partially explaining the differential phenotypes in cells infected by HPV or not, upon Oct4 deregulation.

## Results

### Oct4 is expressed in cervical cancers

**Fig 1:**
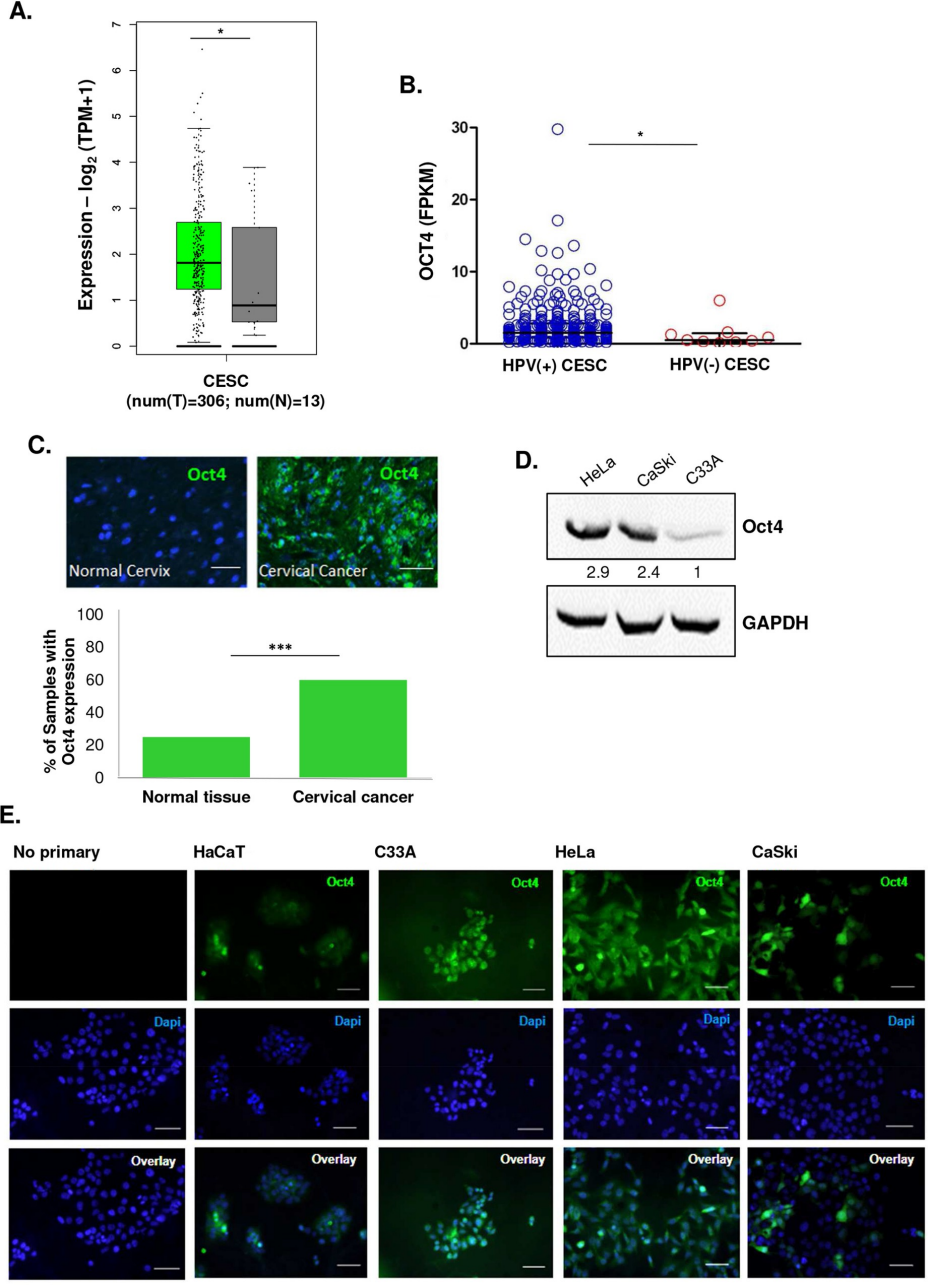
Oct4 is expressed in cervical tumors. (A) Oct4 mRNA levels are higher in cervical cancer (TCGA-CESC, n = 306) compared to normal cervical tissues (GTEx, n = 13) (B) HPV (+) cervical tumors have higher levels of Oct4 transcripts compared to HPV(-) tumors (p<0.05). (C) Analysis of the protein levels of Oct4 from tissues derived from cervices of healthy and cancer patients were made with the use of Tissue Microarrays (TMA). Immunofluorescence revealed Oct4 expression in the majority (31 out of 54) of cervical cancer biopsies (shown in green). (D&E) Oct4 was detected in cervical cancer cell lines via Western Blot and Immunofluorescence (shown in green). HPV(+) (HeLa & CaSki) and HPV(-) (C33A) cell lines express Oct4 (Scale bar, 100μm). Human immortalised keratinocytes (HaCaT) express extremely low levels of Oct4. Mann Whitney analysis was used with *p<0.05, **p<0.01,***p<0.001, ****p<0.0001).

The expression of Oct4 was previously reported in some tumors [6, 8–10, 12], but its role in somatic and cancer cells is still controversial [1–5]. To investigate Oct4 expression in cervical cancer, we extracted Oct4 gene expression data from the Cancer Genome Atlas (TCGA) CESC dataset and compared them to the normalised corresponding levels using normal cervical tissue that was extracted from the GTEx, FANTOM5 and HPA projects (Fig 1A). This analysis showed that Oct4 is upregulated in cervical cancers compared to the normal cervix. Interestingly, Oct4 expression was significantly higher in HPV(+) cervical tumors within the TCGA-CESC dataset (Fig 1B). We wondered whether this pattern extended to other HPV-related cancers, thus we interrogated the expression of Oct4 in HPV(+) and HPV(-) Head and Neck Squamous Cell Carcinoma (HNSCC). We further confirmed that the levels of Oct4 are significantly up-regulated in the HPV(+) than HPV(-) HNSCC matching the effect observed in HPV-associated cervical cancer (S1A Fig). In addition, we noticed that different HPV subtypes, express different levels of Oct4 in cervical cancer. For instance, HPV-16, HPV-18, HPV-45 and HPV-52 subtypes exhibited the highest Oct4 expression levels compared to HPV(-) tumors (S1B Fig). To validate Oct4 expression at the protein level in cervical tumors, we used a tissue microarray containing 54 HPV(+) cervical cancer samples and normal controls. Immunofluorescence revealed an abundant expression of Oct4 among cervical cancer samples (positive signal in approximately 60% of the cancers) (Fig 1C), underscoring the validity of expression differences described in our conclusions from the TCGA data. Since Oct4 was recently shown to play a role in many cancer types, we decided to probe for Oct4 expression in three cervical cancer cell lines (HeLa, HPV18(+), CaSki, HPV16(+), and C33A, HPV(-)). Immunofluorescence analysis revealed that all three cell lines express Oct4, independently of the presence of HPV (Fig 1D and 1E), but higher Oct4 protein levels were detected in the HeLa and CaSki cell lines compared to C33A cells. Human immortalised keratinocytes (HaCaT) used as a control cell line have undetectable expression of Oct4 protein.

### The presence of HPV oncogene products correlates with increased Oct4 expression

**Fig 2:**
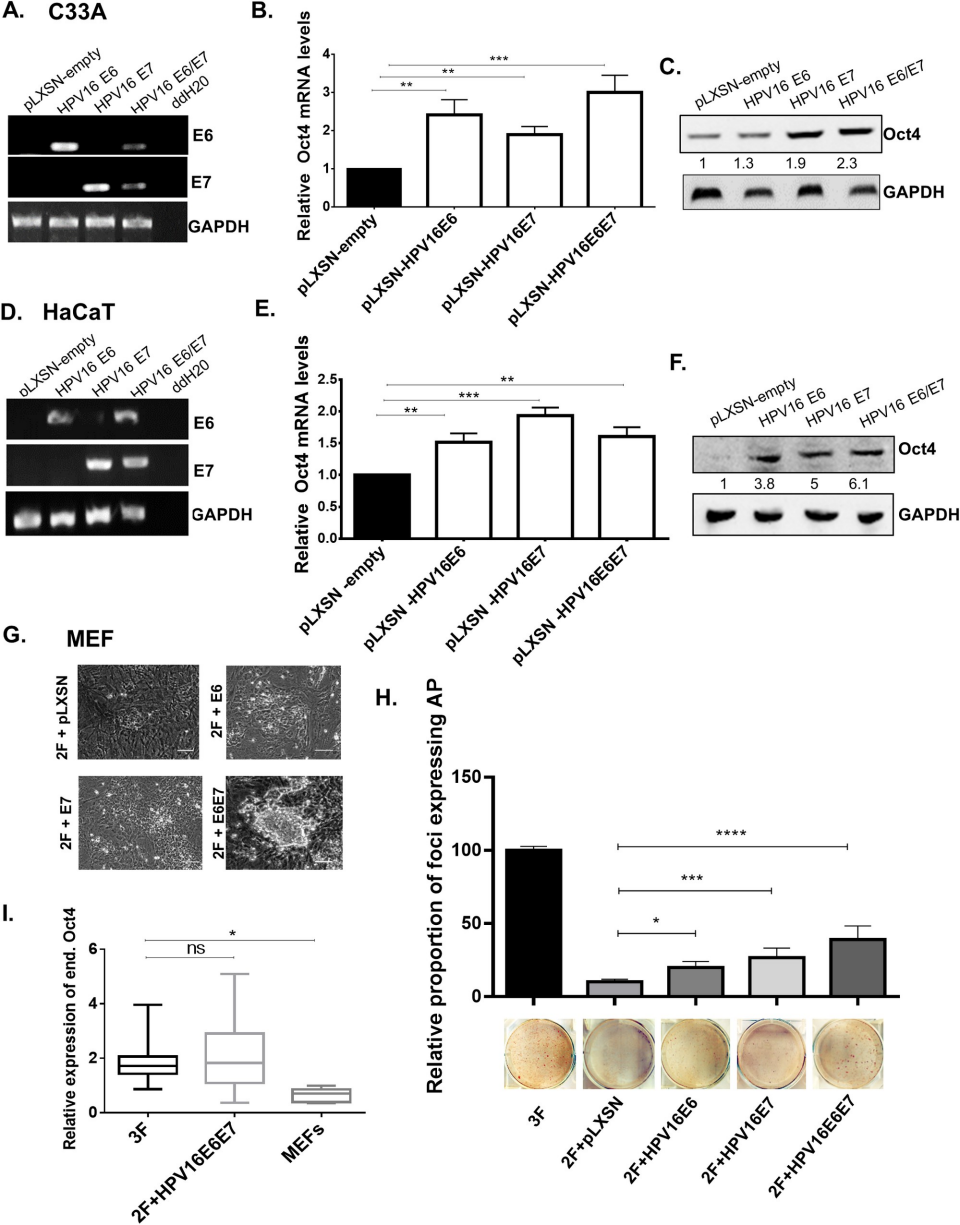
Expression of HPV viral oncogenes results in increased Oct4 expression. HPV(-) cervical cancer cells (C33A) and immortalised human keratinocytes (HaCaT) were transduced with the pLXSN-empty, pLXSN-HPV16E6, pLXSN-HPV16E7 and pLXSN-HPV16E6E7 vectors. (A&D) Successful transduction of cells with HPV16 viral oncogenes is illustrated by RT-PCR using specific E6 and E7 primers. (B-C, E-F) Both the transcript and protein levels of Oct4 in C33A HPV(-) cells and Human immortalised keratinocytes (HaCaT) upon infection with the viral oncogenes are illustrated with the use of qRT-PCR and western blot. All data presented are the mean±SEM of three independent experiments. The statistical test used was unpaired t-test (two-sided) with p<0.05. (ns = non-significant, *p<0.05, **p<0.01, ***p<0.001, ****p<0.0001). (G) Mouse embryonic fibroblasts (MEFs) are reprogrammed when exogenous Oct4 is replaced with both viral oncogenes E6 and E7. (H) The relative proportion of alkaline phosphatase-positive foci formed when Oct4 is replaced with the viral oncogenes is illustrated with a bar chart. Representative images of AP plates used in the quantification are shown. (I) Colonies reprogrammed using 2F+E6E7 (In the absence of exogenously provided Oct4) express endogenous Oct4 at levels comparable to those seen in 3F colonies, shown by qRT-PCR. The data presented are the mean±SEM. The statistical test was one-way ANOVA (two-sided) with p<0.05. (ns = non-significant, *p<0.05, **p<0.01, ***p<0.001, ****p<0.0001).

To interrogate whether the viral oncogenes play a role in the elevated expression of Oct4 in HPV(+) cancer cells, we transduced HPV(-) C33A cells with empty (pLXSN) or HPV16 E6E7-expressing retrovirus, and determined Oct4 levels by qRT-PCR and immunoblotting. Successful transduction of the viral oncogenes in C33A cells was confirmed with semi-quantitative RT-PCR (Fig 2A). We detected an increase in Oct4 transcripts in C33A transduced cells with the viral oncogenes and Oct4 protein levels were elevated in the presence of the E6 and E7 oncogenes (Fig 2B and 2C). This is in agreement with the analysis of the TCGA data (Fig 1B, S1A Fig) showing higher Oct4 expression levels among HPV(+) tumors. To examine whether the viral oncogenes impact Oct4 levels in untransformed cells, we used HaCaT cells that were previously transduced with the two viral oncogenes (Fig 2D). Yet again, the levels of Oct4 were elevated in the HPV16 E6- and E7-transduced HaCaT cells (Fig 2E and 2F). To investigate whether the expression of Oct4 in HaCaT cells increases with E6 and E7 of different HPV subtypes, we transfected HaCaT cells with constructs expressing MSCV-FlagHPV11E6, FlagHPV11E7, FlagHPV16E6, FlagHPV16E7, FlagHPV18E6, FlagHPV18E7 and the control MSCV-FlagGFP. Two days post-transfection HaCaT cells were collected. Semi-quantitative PCR was performed to validate successful transfection of the HPV-expressing constructs. We measured Oct4 transcript and protein levels using qRT-PCR and western blot, respectively. We found that HaCaT cells expressing either E6 or E7 from different HPV subtypes, express moderately higher levels of Oct4 regardless of subtype (S1C–S1E Fig).

We wondered whether HPV16 E6 and E7 can functionally impinge on processes involving Oct4 and to assess that we utilised cellular reprogramming assays. The traditional reprogramming cocktail originally included the transcription factors Sox2, Klf4, and Oct4 [3], a fourth factor c-Myc was later shown to be dispensable [22]. In MEFs, all three factors are required and reprogramming has not been achieved in the absence of Oct4 without exogenous complementation. We replaced exogenously-provided Oct4 with a retrovirus expressing the HPV16 oncogenes. Surprisingly, MEFs were reprogrammed in the absence of exogenous Oct4 (2F: exogenous expression of only pMXsSox2, and pMXsKLF4), only when both E6 and E7 were also expressed further implicating the viral oncogenes in the circuitry which regulates Oct4 expression. When only retroviruses expressing Sox2 and Klf4 (2F) along with pLXSN control, pLXSNE6 or pLXSNE7 were expressed in MEFs, no iPS-like colonies were obtained (Fig 2G). When the absence of Oct4 was complemented with E6 and E7 (pMXsSox2, pMXsKLF4, pLXSNE6E7), iPS-like colonies appeared, albeit at a lower frequency compared to reprogramming with all three factors. Early reprogramming was also quantified using chromogenic alkaline phosphatase (AP) assays, an early marker for reprogramming. The abundance of foci with AP expression generally correlated to the appearance of morphologically distinguishable iPS colonies (Fig 2H). However, it is important to note that AP expression is an early event in reprogramming and **small foci of expression do not necessarily correspond to the formation of morphologically distinguishable colonies.** Even though a slight increase in AP-positive colonies was observed in the 2F+pLXSN, 2F+HPV16E6, and 2F+HPV16E7 conditions, compared to untransduced MEFs, morphologically distinguishable iPS colonies were only apparent in 2F+HPV16E6E7 condition. Importantly, all reprogrammed colonies isolated expressed comparable levels of endogenous Oct4, suggesting that E6 and E7 contribute to the process by participating (directly or indirectly) to the activation of the endogenous Oct4 locus (Fig 2I).

### Oct4 promotes the proliferation and migration of HPV(-) cervical cancer cells

**Fig 3:**
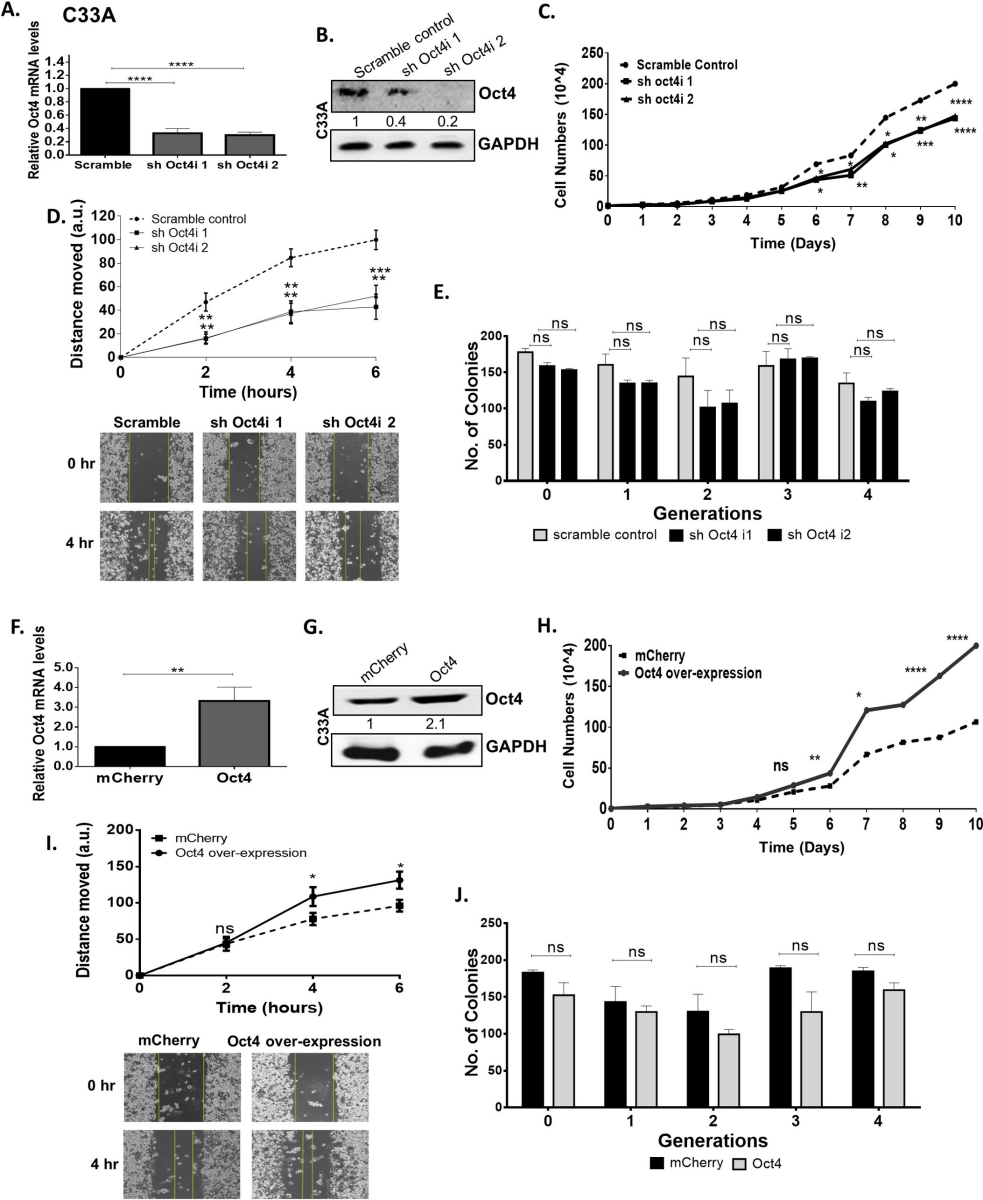
Oct4 promotes proliferation and migration in HPV-negative cells. Short-hairpin RNA was used to downregulate Oct4 from C33A cells. (A&B) Both Oct4 transcript and protein levels were successfully depleted as indicated with qRT-PCR and western blot. Significance was calculated using two-sided unpaired t-test. (C) A cell growth curve was constructed to assess cell numbers following stable Oct4 knockdown. (D) Cell motility of serum-starved Oct4-knockdown C33A cells was examined via the use of in-*vitro* wound healing assay at 2, 4, and 6 hours post wounding. Representative images of the wounds are shown. (E) The ability of Oct4-knockdown C33A cells to form tumorpheres and self-renew was indicated via the tumorsphere formation assay. (F&G) An inducible system to upregulated Oct4 in C33A cells was set up and upon Oct4 overexpression, C33A cells gained the ability to (H) proliferate and (I) migrate faster compared to the mCherry control condition whereas (J) their self-renewing capacities did not change. Scale bars are 100μm. All the data shown are mean±SEM of three independent experiments. The statistical test used was Mann-Whitney U t-test (two-tailed) (ns = non-significant, *p<0.05, **p<0.01, ***p<0.001, ****p<0.0001).

To investigate the *in vitro* biological significance of Oct4 in HPV(-) cervical cancer cells, we generated stable Oct4-knockdown C33A cells using short-hairpin RNA vectors. We confirmed that both the relative mRNA (Fig 3A) and protein levels of Oct4 decreased compared to the scramble control (Fig 3B and S2A Fig).

To examine whether Oct4 affects the proliferation of HPV(-) C33A cells we generated a growth curve, for a period of 10 days, of Oct4-knockdown and control cells. We found that Oct4-knockdown C33A cells attained a proliferative disadvantage compared to the scramble control (Fig 3C). Cell cycle analysis of C33A Oct4-knockdown and control cells was performed to determine in which phase of the cell cycle the cells are mostly accumulated. Propidium iodide stain was used to differentiate between cell cycle stages. We observed that upon Oct4 knockdown there is a higher proportion of the cells arrested in the G1-phase compared to the control cells, validating the outcome obtained from the growth curves (S3B Fig).

To address the impact of Oct4 in cell migration, wound healing assays were performed. To avoid confounding of migration outcomes due to proliferation differences, cells were serum-starved in order to abrogate proliferation (S4A Fig). Migration was measured at times when proliferation was unaffected. We found that Oct4-knockdown levels in serum starved HPV(-) cells led to impaired migration (Fig 3D).

Oct4 expression was previously linked to cancer stem cell activity [23]. Recurrent episodes of cancer are associated with cancer stem cells, which have the ability to self-renew hence, we sought to investigate the impact of Oct4 in the formation of cervical tumorspheres, a proxy for the potential of cells to exhibit stem cell traits [24]. HPV(-) cells with stable expression of Oct4 knockdown were used to explore the effect of Oct4 on cancer cell clonogenesis. The tumorsphere-forming capacity of Oct4-knockdown C33A cells displayed no significant change when compared to control cells over 4 serial passages (Fig 3E).

Conversely, to further examine the impact of Oct4 in HPV(-) cells, we overexpressed the protein in C33A cells. Real-time quantitative PCR and western blot validated the increase in the transcript and protein levels of Oct4 (Fig 3F and 3G, S2B Fig). We noticed that upon Oct4 overexpression, C33A cells gained a proliferative and migratory phenotype compared to the mCherry control cells. These phenotypic changes are opposite to the Oct4-knockdown phenotypes. Regarding the tumorsphere capacities of Oct4-knockdwon C33A and control cells, no significant changes were obtained over four passages (Fig 3H–3J).

### Oct4-mediated phenotypes vary depending on the HPV status of cervical cancer cells

**Fig 4:**
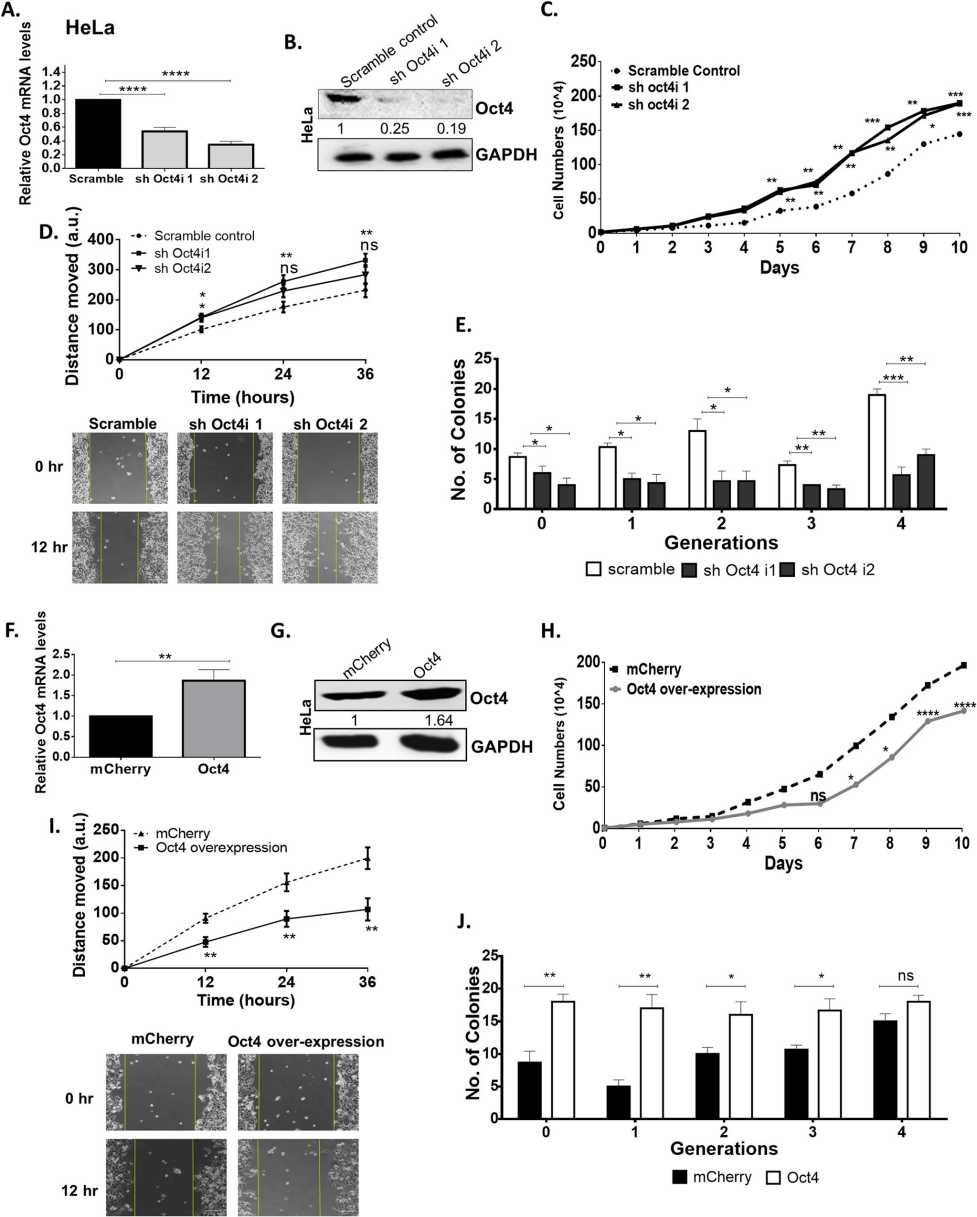
Oct4 attenuates proliferation, migration in HPV18 HeLa cells. (A & B) HPV18 HeLa cells were subjected to stable Oct4-knockdown verified with qRT-PCR and Western blot. Significance was calculated using unpaired t-test (two-tailed). A comparison between HeLa Oct4-knockdown and control cells was made while assessing (C) proliferation (D) migration (12–36 hours post-wounding) and (E) tumorsphere formation. (F&G) Stable Oct4 overexpression in HeLa cells was performed and verified with qRT-PCR and Western blot. (H-J) HeLa cells expressing the Oct4 overexpression exhibited a decreased proliferative and migratory phenotype while their sphere-forming capacity increased compared to the control. Scale bars are 100μm. The data shown are the mean±SEM of three independent experiments. (Mann-Whitney U t-test, two-tailed) (ns = non-significant, *p<0.05, **p<0.01, ***p<0.001, ****p<0.0001).

**Fig 5:**
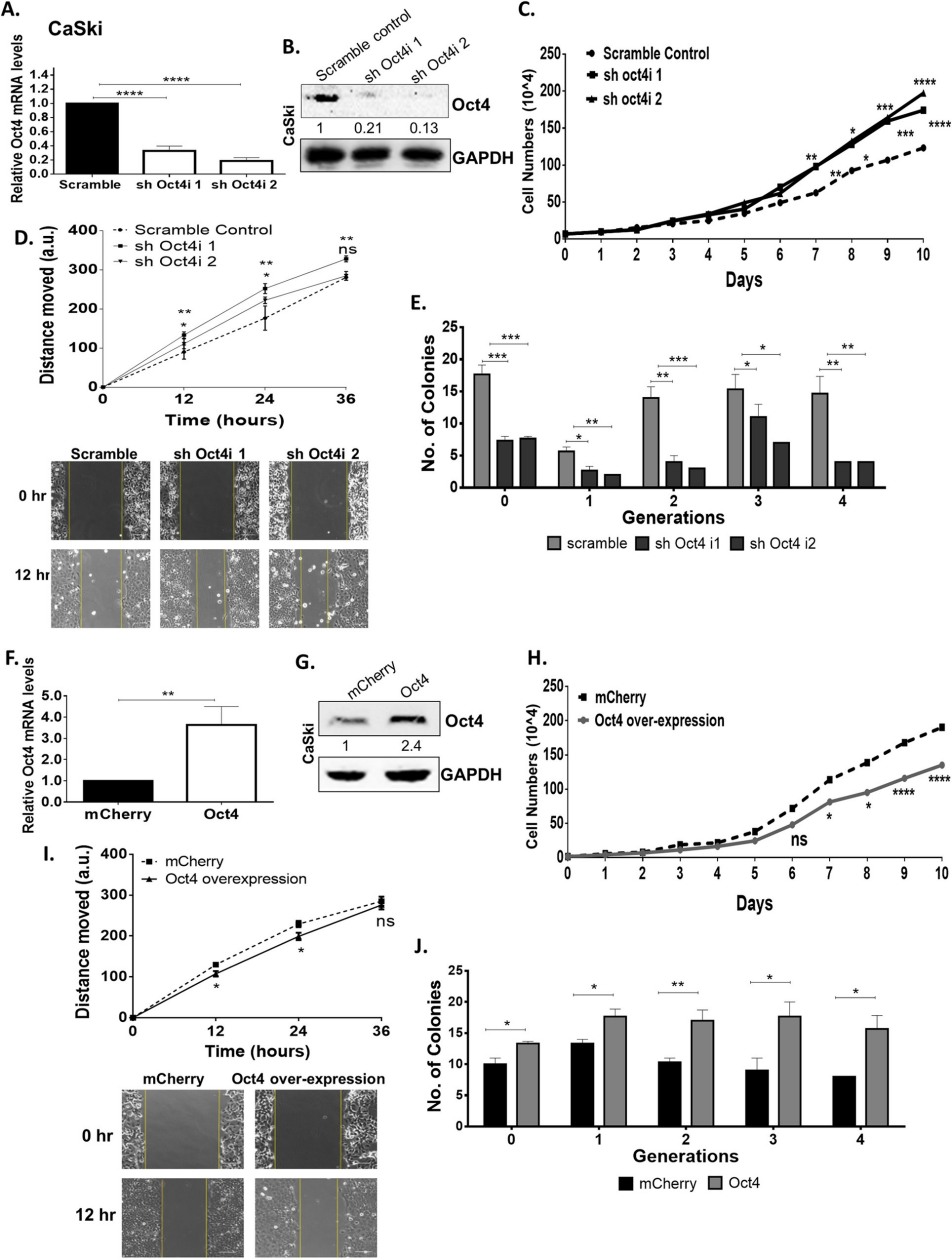
Oct4 attenuates proliferation and migration while augments self-renewal in HPV16 CaSki cells. (A & B) HPV16 CaSki cells were subjected to stable oct4-knockdown verified with qRT-PCR and Western blot (Significance was calculated using unpaired t-test (two-tailed)). A comparison between Oct4-knockdown CaSki cells and their scramble control was made while assessing (C) proliferation (D) migration (12–36 hours after the gap formation) and (E) tumorsphere formation. (F&G) Stable Oct4 overexpression in CaSki cells was performed by using an inducible Oct4 construct and mCherrry was used as a control. (H-J) Oct4-overexpressed CaSki cells exhibited a decreased proliferative and migratory pattern but displayed an elevated tumorsphere formation capacity compared to the control. Scale bars are 100μm. The data shown are the mean±SEM of three independent experiments. Mann-Whitney U t-test (two-sided) was used (ns = non-significant, *p<0.05, **p<0.01, ***p<0.001, ****p<0.0001).

To examine the effects of Oct4 in HPV(+) cancers, HeLa and CaSki cells were subjected to Oct4-knockdown. We validated that both the relative mRNA (Figs 4A and 5A) and protein (Figs 4B and 5B and S2A Fig) levels of Oct4 decreased by 60% in the HPV(+) cervical cancer cell lines compared to the scramble control. To investigate whether Oct4 affects cell proliferation, we generated cell growth curves over a period of 10 days. We noticed that Oct4-knockdown HPV(+) cells (HeLa and CaSki) attained a proliferative advantage over the scramble control (Figs 4C and 5C). Cell cycle analysis was performed in these cells to quantify the proportion of cells accumulated at each phase of the cell cycle. We found that more cells are accumulated in the S- and G2/M phase of the cell cycle upon Oct4-knockdown compared to the control cells. (S3A Fig). To assess the impact of Oct4 overexpression on the proliferation of HPV(+) cervical cancer cells over a period of 10 days, cells were counted and plotted in the form of growth curves. Our analysis showed that Oct4-overexpressing HeLa and CaSki cells demonstrate decreased cell proliferation (Figs 4H and 5H). Thus, we conclude that while Oct4 promotes proliferation in HPV(-) cells as described for other cell types, it inhibits proliferation in HPV(+) cells.

Oct4 has previously been implicated in cancer cell migration [12]. To probe potential roles for Oct4 in the migration of cervical cancer cells, we conducted wound healing assays. Oct4 knockdown in HPV(-) cells led to impaired migration as mentioned above, whereas the opposite trend was observed in HPV(+) cells (Figs 4D and 5D). Genes involved in migratory pathways such as the actin isoform ACTB and LIM domain kinase 1 (LIMK1), a regulator of actin polymerization, are found to be affected by the Oct4 knockdown. The deregulation of these genes validate the migration pattern revealed by the wound healing assay in HPV(+) and HPV(-) cell lines (S4B Fig). As expected, Oct4 overexpression in cervical cancer cells yields the opposite phenotype in cell motility as seen in Oct4 knockdown. Oct4 overexpression led to impaired migration in HPV(+) HeLa and CaSki cells, however CaSki cells displayed more modest differences compared to the control cells (Figs 4I and 5I).

To study the tumorsphere-forming capacities of HPV(+) cells upon Oct4 deregulation, stable Oct4-knockdown HeLa and CaSki cells were used. Upon Oct4 knockdown the cells demonstrated an impaired ability to form tumorspheres over 4 serial passages (Figs 4E and 5E). In contrast, Oct4 overexpressing HPV(+) cells exhibited an enhanced tumorsphere-forming capacity compared to the control cells (Figs 4J and 5J). Next, we performed qRT-PCR to examine whether cervical tumorspheres being formed in response to Oct4 overexpression, exhibit an enhanced expression of stemness markers. Our results show that Oct4, as well as other stemness markers such as Sox2 and Klf4, are indeed enriched in the cervical tumorspheres compared to the cancer cell monolayers and this was true in all conditions tested (S5 Fig).

### Oct4 expression in E6/E7-transduced human immortalised keratinocytes mimics Oct4-mediated phenotypes in HPV(+) cells

**Fig 6:**
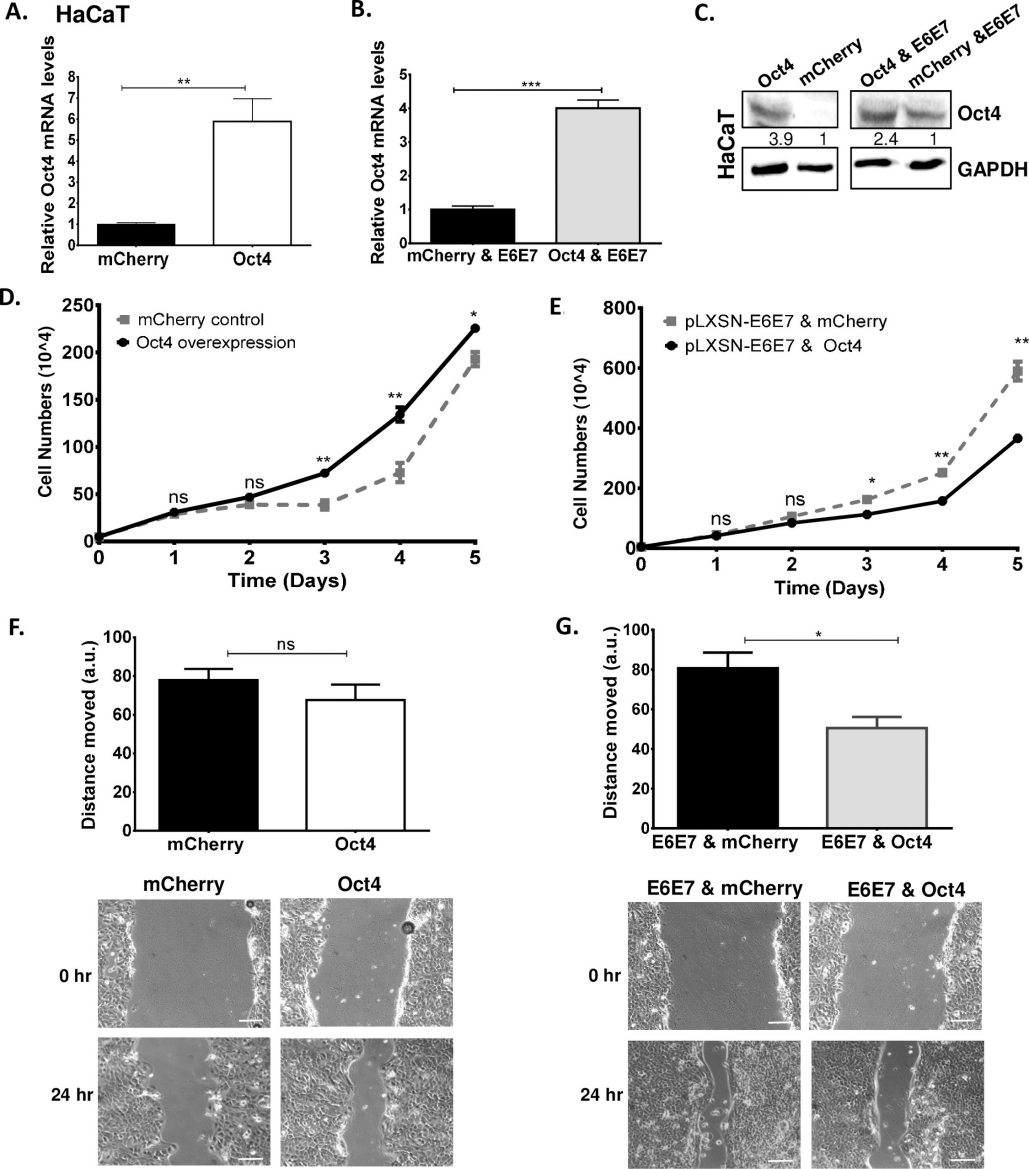
Oct4 in association with the viral oncogenes reduces the ability of human immortalised keratinocytes to proliferate and migrate. Human immortalized keratinocytes (HaCaT) were transduced with Oct4 and HPV16 E6E7 constructs. (A-C) The validation of Oct4 transduction in HaCaT Cells is shown with qRT-PCR and Western blots. Unpaired t-test (two-tailed) was used to calculate significance. Growth curves to assess proliferation in HaCaT cells transduced with (D) Oct4 and mCherry control and (E) Oct4 & pLXSN-HPV16E6E7 and mCherry & pLXSN- HPV16E6E7 were shown. (F&G) Wound healing assays exhibited the ability of HaCaT transduced cells to migrate. Representative images of the scratch assay are shown. Scale bars are 100μm. The data shown are the mean±SEM of three independent experiments. Mann-Whitney U t-test (two-sided) was used (ns = non-significant, *p<0.05, **p<0.01, ***p<0.001, ****p<0.0001).

To address the effect of Oct4 in non-transformed cells, HaCaT cells were lentivirally transduced with an inducible Oct4 construct. The upregulated transcript and protein levels of Oct4 were verified with qRT-PCR and Western blot (Fig 6A and 6C). We then interrogated the impact of Oct4 on keratinocyte proliferation. High Oct4 expression in keratinocytes was associated with increased proliferation (Fig 6D). To interrogate whether the viral oncogenes of HPV can modulate the activity of Oct4, we transduced HaCaT cells with pLXSN-HPV16 E6E7. Then E6/E7- transduced HaCaT cells were subjected to stable Oct4 overexpression and the elevated Oct4 mRNA and protein levels were confirmed (Fig 6B and 6C). A growth curve was constructed to examine the proliferation of E6/E7-Oct4 transduced HaCaT cells. We found that in the presence of E6/E7 and Oct4, cell proliferation is reduced compared to control cells (Fig 6E). These data are in agreement with the Oct4-mediated proliferation in HPV(+) cells supporting the conclusion that Oct4 expression in the context of viral oncogene expression has a distinct impact on proliferation (opposite to that observed in the absence of the oncogene products).

To determine the impact of Oct4 and E6/E7 on the motility of HaCaT cells, wound healing assays were carried out. We found that in HaCaT cells, Oct4 does not significantly affect the migration of keratinocytes compared to the control at 24 hours post-wounding. However, when Oct4 and the viral oncogenes are expressed together in keratinocytes, migration is attenuated (Fig 6F and 6G). These results corroborate that the Oct4-associated phenotypes in HPV(+) cancer cells are linked specifically to the presence of the HPV oncogenes.

### Transduction of HPV(-) C33A cells with E6/E7 mirrors the transcriptional program and Oct4-mediated proliferation of HPV(+) cells

**Fig 7:**
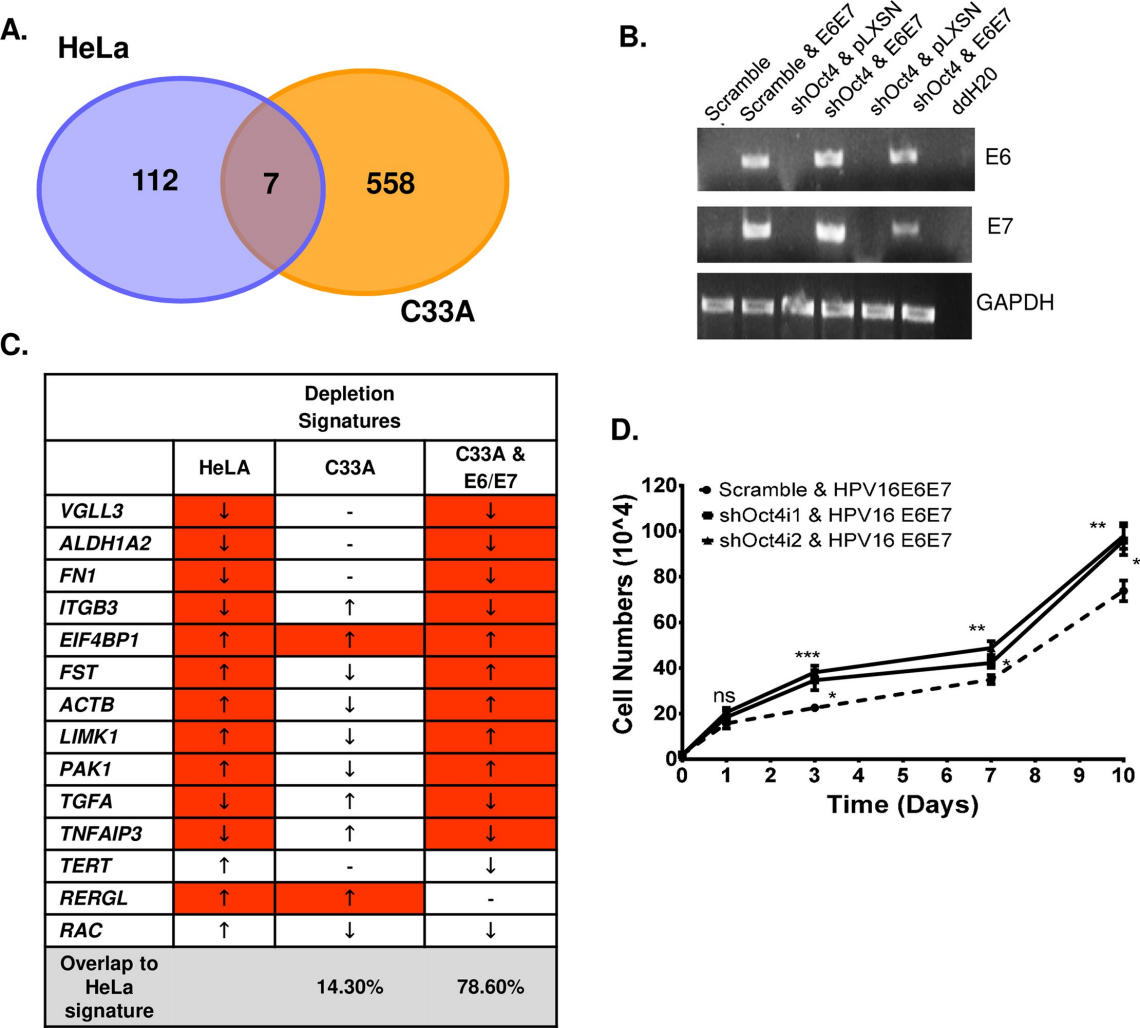
Addition of the viral oncogenes in HPV-negative C33A cells leads to proliferation and transcriptional rescue. RNA-sequencing analysis revealed differentially expressed genes in HPV-positive (HeLa) and–negative (C33A) cells. (A) Amongst the highly deregulated genes, only seven are shared in HeLa and C33A cells. (B) Semi-quantitative PCR shows successful infection of the HPV16 viral oncogenes in stable Oct4-knockdown C33A cells. (C) 78.6% of the genes that were deregulated in Oct4-depleted C33A-E6E7 cells adopt a profile resembling that of HeLa Oct4-depletion signature. A two-sided Unpaired t-test was used to calculate significance (D) Cell numbers of Oct4-knockdown C33A cells upon transduction with the viral oncogenes at 1, 3, 7 and 10 days are illustrated in the form of growth curve. Statistical differences were examined by Mann-Whitney U t-test (two-tailed). All the data plotted are taken from three independent experiments (mean±SEM), (ns = non-significant, *p<0.05, **p<0.01, ***p<0.001, ****p<0.0001).

To gain an insight on the impact of the viral oncogenes on Oct4-mediated phenotypes, we subjected HeLa-HPV(+) and C33A-HPV(-) cell lines to transcriptome sequencing. Importantly, there were just a few co-deregulated genes (up- or down-) between the two cell lines upon Oct4 knockdown (*SPOCK2*, *KCTD12*, *LENG1*, *HSPA1A*, *NPIPA5*, *ADRA2C*, *FAM45BP*) (Fig 7A). These data, support that the top upregulated genes in these two cell lines, participate in different biological processes and have different molecular functions, in the presence or absence of HPV (S6 Fig). We further confirmed the anticipated expression levels of at least 14 significantly deregulated genes, in C33A and HeLa Oct4 knockdown cells, by qRT-PCR (S7A–S7D Fig). We used these highly deregulated genes upon Oct4 knockdown to define the “**Oct4-depletion signatures”** for HeLa and C33A cells respectively (S7 Fig).

To understand whether changes in the transcriptome between the two different cell lines upon Oct4 knockdown, are partly due to the presence of the E6/E7 oncogenes and not merely a result of differences in genetic background, we generated stable C33A-E6E7 cells by transducing C33A cells with retroviruses expressing the HPV16 oncogenes (Fig 7B). Oct4 was then knocked-down in these cells and the expression of genes collectively called the “HeLa Oct4-depletion signature” (total of 14 genes), was assessed using qRT-PCR. This comparison revealed that in Oct4-knockdown C33A cells, only 14.3% (2 out of 14) of the genes matched the profile of the HeLa Oct4-depletion signature; whereas in Oct4-knockdown C33A-E6E7 cells the percentage of the genes corresponding to the HeLa Oct4-depletion signature, increased to 78.6% (11 out of 14) (Fig 7C). Furthermore, Oct4-knockdown C33A-E6E7 cells only shared the C33A Oct4-depletion signature by 28.6% (4 out of 14 genes) (S7E–S7G Fig). Importantly, attenuated proliferation seen in C33A cells upon Oct4 downregulation was reversed in the context of E6 and E7 expression (Fig 7D), confirming that the presence of the viral oncogene products modifies both the transcriptional output, as well as related phenotypes mediated by Oct4 in these cells.

### HPV E7 interacts with endogenous Oct4

**Fig 8:**
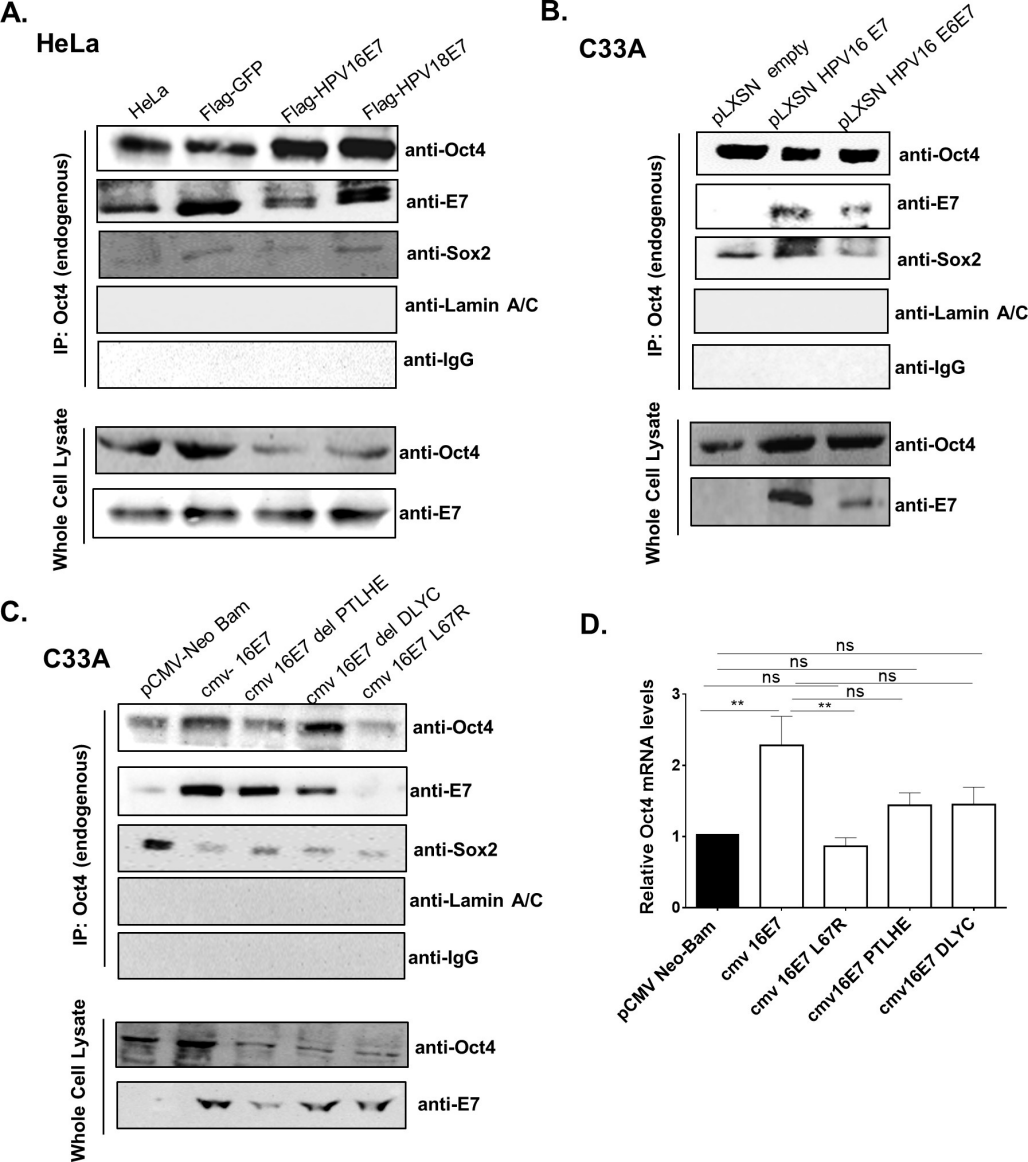
Oct4 interacts with HPV E7. (A) Co-immunoprecipitation analysis was performed in HeLa cells transduced with FLAG-tagged GFP, HPV16-E7 and HPV18-E7 vectors. Endogenous Oct4 was immunoprecipitated with IgG sepharose-beads and Oct4 interactors were revealed with Western blot. Antibodies for Sox2 and LaminA/C were used as a positive and negative control for Oct4 interaction respectively. IgG-antibody was used as a negative control for the immunoprecipitation experiment. (B) C33A cells transduced with either pLXSN-E7, pLXSN-E6E7 and pLXSN-empty constructs were used. Endogenous Oct4 was immunoprecipitated and interactions were visualised via western blot. Endogenous Oct4 interacts with E7. (C) C33A cells transfected with Mutants from the CR1 (del PTLHE), CR2 (del DLYC) and CR3 (L67R) domains of the E7 protein were used to test interaction with endogenous Oct4 (D) C33A cells were either transfected with pCMV-neo bam empty, cmv 16E7 wildtype or cmv 16E7 mutants. Cells were collected 48-hours post transfection and RNA was extracted for the analysis of Oct4 mRNA levels with qRT-PCR. Three independent replicates (mean±SEM) were used and statistical analysis was performed with Unpaired t-test (two-tailed) (ns = non-significant, *p<0.05, **p<0.01, ***p<0.001, ****p<0.0001).

The differential effects of Oct4 regulation in HPV(+) and HPV(-) cells raise the possibility that the HPV oncogenes interfere with its activity. An interaction between Oct4 and HPV E7 was previously reported, but the extent to which this happens in physiologically relevant cells is still unknown [25]. To interrogate a potential physical interaction between E7 and Oct4, HeLa cells were retrovirally transduced with E7 from “high-risk” HPV subtypes (HPV16 and HPV18) and were then harvested and subjected to immunoprecipitation analysis. E7 was found to precipitate with endogenous Oct4 in HeLa cells (Fig 8A). We validated the Oct4-E7 interaction by transducing pLXSN- empty control, pLXSN-HPV16E7 and pLXSN-HPV16E6E7 vectors in HPV(-) C33A cells (Fig 8B). To map the site of the Oct4 interaction on the E7 protein we used mutants from the CR1, CR2 and CR3 regions of HPV16 E7. Specifically, we used the deletion mutants cmv-16E7 del PTLHE (del 6-10aa of the CR1 region) and cmv-16E7 del DLYC (del 21–24 of the CR2 region) and a CR3 mutant which has a point mutation on the hydrophobic core of the E7 protein the L67R. C33A cells were transfected with an empty pCMV-vector, cmv-HPV16E7 and the three E7 mutants and then we immunoprecipitated endogenous Oct4. Surprisingly, the CR3 mutant L67R completely failed to bind Oct4 whereas Oct4 retained the ability to bind E7 with the PTLHE and DLYC mutants (Fig 8C). This constitutes the first demonstration that the binding of Oct4 to E7 is confined to the CR3 region of the protein. To exclude the possibility of non-specific binding to Oct4 we performed a mock-IP by transfecting C33A cells with cmv-16E7, cmv-16E7 L67R and GFP-expressing constructs. Then, we immunoprecipitated GFP by using a GFP antibody and blotted for Oct4. No interaction with GFP was detected, further supporting the conclusion that the Oct4-E7 interaction is specific. (S8D Fig). Of note, the transcript levels of Oct4 in the presence of the E7 mutants are not elevated compared to the empty control, unlike in the presence of the wildtype HPV16 E7 (Fig 8D). This would suggest that the E7-mediated upregulation of Oct4 is a separate activity from its ability to bind Oct4 and may be linked to the pRb-binding domain of E7.

### Oct4-related proliferation in keratinocytes and cancer cells is mediated by the Oct4-E7 interaction

**Fig 9:**
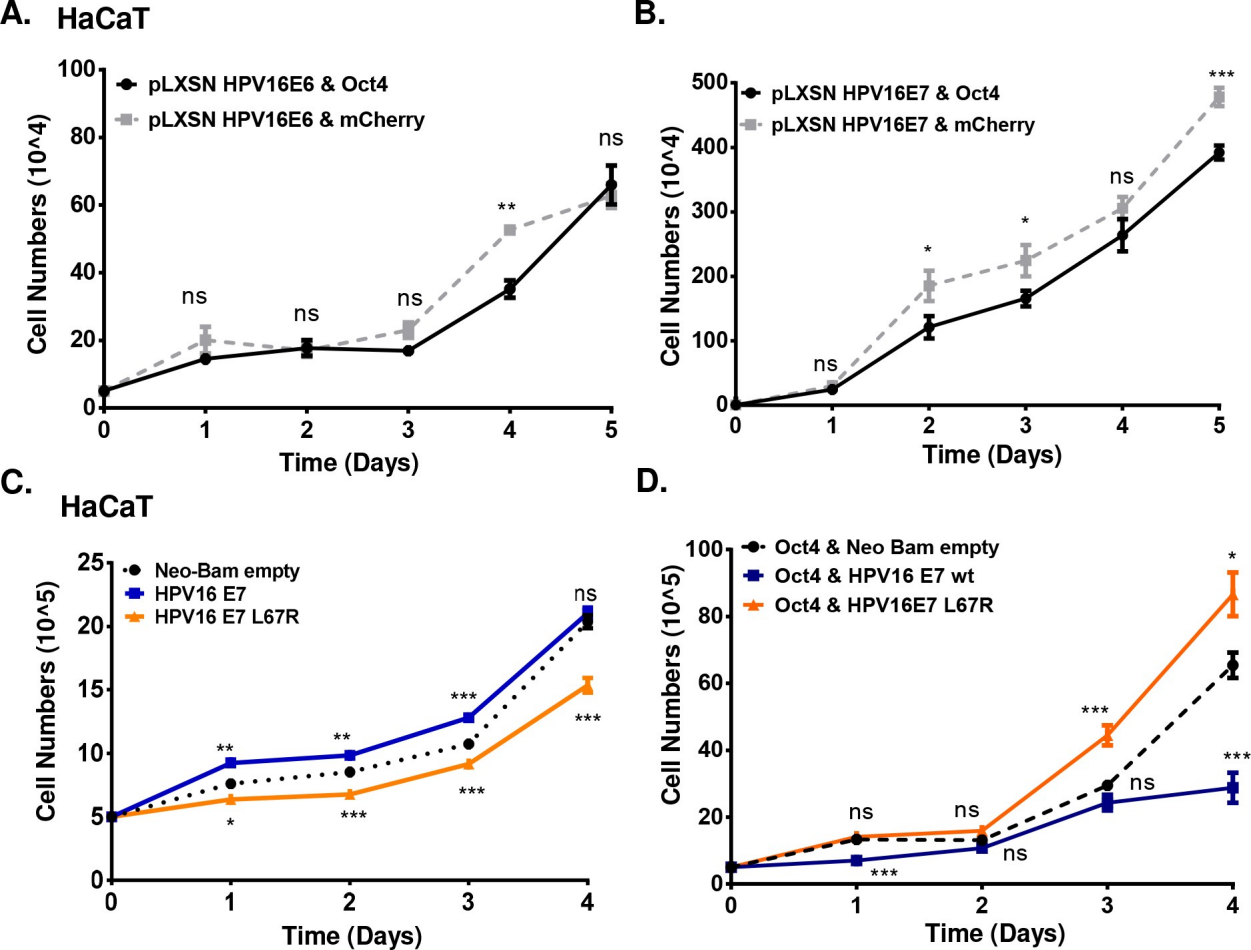
HaCaT Oct4-related proliferation is mediated by the Oct4-E7 interaction. HaCaT cells with stable expression of (A) pLXSN HPV16E6 & Oct4 and pLXSN HPV16E6 & mCherry (B) pLXSN HPV16E7 & Oct4 and pLXSN HPV16E7 & mCherry were used to construct a growth curve for a period of 5 days. (C) HaCaT cells were transfected with cmv-Neo Bam empty, cmv 16E7 wildtype and cmv 16E7 L67R constructs. The proliferation of those cells was presented in the form of a growth curve. (D) Stable Oct4-transduced HaCaT cells were also transfected with cmv-Neo Bam empty, cmv 16E7 wildtype and cmv 16E7 L67R constructs to examine the effect of Oct4 and E7 on the proliferation of immortalized keratinocytes. The p value shown on the graph indicates the comparison between the wildtype and mutant E7 with the empty control. The comparison of wildtype E7 with the L67R mutant indicates of p value of (Day 1 ****p<0.0001, day 2 **p<0.01, day 3 ***p<0.001, day 4 ****p<0.0001). Data were derived from three biological replicates each done in triplicate (mean±SEM) and statistical analysis was performed with Mann-Whitney U t-test (two-sided) (ns = non-significant, *p<0.05,**p<0.01,***p<0.001,****p<0.0001).

To dissect the individual contribution of E6 and E7 to Oct4-mediated phenotypes we used HaCaT cells transduced with Oct4 and pLXSN-HPV16 E6 or pLXSN-HPV16 E7. Growth curves have been constructed over a period of 5 days as presented in Fig 9. We detected minimal changes in proliferation upon co-expression of E6 and Oct4 compared to E6 alone (difference only in day 4) (Fig 9A). In contrast, co-expression of E7 and Oct4 led to attenuated proliferation compared to E7 expression alone (Fig 9B). This result is comparable with Oct4-mediated phenotypes upon E6 and E7 co-expression (Fig 6E) suggesting that the effect of Oct4-related proliferation in HPV(+) cells and HaCaT cells expressing E6/E7-Oct4 is largely attributable to the presence of E7. Semi-quantitative RT-PCR was used to detect the E6 and E7 transcripts in HaCaT cells (S8A Fig) and Western blot was conducted to corroborate the upregulation of Oct4 (S8B Fig). To understand the functional contribution of Oct4-E7 interaction on cellular proliferation and to examine the extent to which the loss of binding of E7 to Oct4 with the E7 L67R mutant impacts proliferation, we transfected HaCaT cells with cmv-Neo Bam empty, cmv-16E7 and cmv-16E7 L67R and assessed proliferation in the absence and presence of Oct4. We found that upon expression of wildtype E7 in HaCaT cells, the proliferation increases compared to the control over a period of 4 days. Nonetheless, proliferation of HaCaT cells diminishes when the L67R mutant is transfected to HaCaT cells (Fig 9C). To interrogate the impact of the loss of binding of E7 to Oct4 via the L67R mutant, we used HaCaT cells transduced with Oct4 and we transfected them with cmv-Neo Bam empty, cmv-16E7 and cmv-16E7 L67R constructs. We determined that E7 expressing HaCaT-Oct4 cells show a modest reduction in proliferation (days 1,4) compared to the control cells, whereas E7 L67R expressing HaCaT-Oct4 cells proliferate slightly faster (days 3, 4). Direct statistical comparison of E7 HaCaT-Oct4 cells versus E7 L67R expressing HaCaT-Oct4 cells was found to be statistically significant through the time-course of the experiment (Fig 9D). This suggests that the interaction of E7 with Oct4 is important for the attenuation of cellular proliferation. It is worth noting that the differences observed in proliferation in this specific cell type in the presence of both wildtype E7 and the L67R mutant, do not correlate to the levels of Oct4 which increase in the presence of both the wildtype E7 and L67R mutant (S8C Fig).

To further determine whether the role of E7 may be relevant in Oct4-mediated transcriptional output, Oct4-knockdown and scramble-control C33A cells were transfected with wildtype E7 using the cmv-16E7 plasmid. Then the expression profile of the genes (*VGLL3*, *ALDH1A2*, *FN1*, *ITGB3*, *TERT*, *RERGL*, *FST*, *EIF4BP1*, *TGFA*, *TNFAIP3*, *LIMK1*, *RAC*, *PAK1*, *ACTB*) previously identified in Fig 7C was tested with qRT-PCR. We found that 7 out of 14 genes mimic the C33A-E6E7 signature (S8E Fig). Interestingly, upon transfection of the L67R E7 mutant the expression changes of 6 out of 14 genes were opposite to those seen with E7 wildtype expression (S8F Fig).

## Discussion

Oct4 expression is reported in many cancer types including cervical tumors [13, 26]. We confirmed the up-regulation of Oct4 in cervical cancer tissues and cancer cells and we provide additional evidence that Oct4 is upregulated in HPV(+) tumors compared to HPV(-) ones. We also verified a causal relationship between the viral oncogenes and increased Oct4 levels in human immortalised keratinocytes and in the HPV(-) C33A cells, upon infection with the E6/E7 oncogenes [27]. Hence, we hypothesise that both viral oncogenes affect the transcriptional program that regulates the endogenous expression of Oct4 in a direct or indirect manner. The exact mechanism through which the HPV oncogenes promote this upregulation, is not currently understood. Increased proliferation may lead to increased Oct4 levels in an Rb-dependent manner [18], thus we cannot exclude the possibility that the upregulation observed is linked to increased proliferation. Nevertheless, our results would suggest that while E7-mediated transcriptional upregulation may be linked to its pRb binding domain (Fig 8D), changes in proliferation mediated by E7 in the context of Oct4 are linked to the CR3 domain of the protein, thus represent distinct events to the upregulation of Oct4.

The reactivation of Oct4 even in non-transformed cells is remarkable since it is clear that Oct4 is a transcription factor with stringent expression in early development and no detectable expression in most adult healthy tissues. For example, in contrast to Sox2 [28], Oct4 has no known roles in adult stem cells [4]. Nevertheless, Oct4 has recently been implicated in atherosclerosis, where its expression plays an atheroprotective role [5].

Supporting evidence that E6/E7 expression can upregulate Oct4, comes from the complementation of exogenously provided Oct4 with the viral oncogenes in the process of cellular reprogramming. In the past, Oct4 has been replaced in reprogramming assays by genetically expressing factors upstream of Oct4 activation, target genes or by epigenetically activating the Oct4 locus, etc [29, 30]. The formation of iPS colonies is likely due to upregulation of endogenous Oct4, which is detectable in iPS colonies reprogrammed without exogenously provided Oct4, in the presence of E6 and E7. A recent paper claims that highly proliferative cells can be reprogrammed using a cocktail of transcription factors that excludes Oct4 (but includes cMyc) [31]. However, this claim is based on data which show a **correlation** between proliferation and reprogramming efficiency, as well as data where cells are immortalized with SV40LT (similar to E6/E7 expression in some respects). Our findings are consistent with literature and agree with evidence that while proliferation is a necessary co-factor for reprogramming, no conditions have been demonstrated yet where increased proliferation is **sufficient** [32] to replace any factor during reprogramming. We propose that E7 contributes to reprogramming in part by upregulating the expression of Oct4, and increasing proliferation, but also in other ways currently incompletely understood. E7-mediated effects are insufficient to produce morphologically distinguishable colonies but additional functions provided by E6 may contribute to the results seen. While we do not claim that this is reflective of the abilities of the oncogenes to contribute to the formation of stem cells *in vivo*, it provides additional evidence that the oncogenes can impinge on pathways which can lead cells to acquire some stem cell traits. Such activity can be more clearly linked to the oncogenic potential of E6 and E7, however it is unclear whether it also has implications in the role of E6 and E7 during the lifecycle.

Our work demonstrates that Oct4 expression impacts diversely on HPV(+) and HPV(-) cervical tumors. Oct4 has previously been reported to accelerate proliferation in cancer cells [10,33–34]. In cervical cancer cells tested here, we observed a similar effect of Oct4 on proliferation but only in the HPV(-) cell line C33A. In these cells, Oct4 knockdown led to a G1-phase arrest, validating the proliferative disadvantage that was evident in the growth curve. To our surprise, in HPV(+) cancer cell lines we found Oct4 to be a negative regulator of proliferation, its knockdown leading to increased proliferation. Supporting this finding, we also saw an accumulation in the G2/M phase of the cell cycle in Oct4-depleted HPV(+) cells. Inverse trends in Oct4 mediated phenotypes were also noted in cell migration. The differences in migration were also validated by changes in gene transcription patterns. Specifically, genes involved in the actin cytoskeleton pathway were found to be deregulated matching the Oct4-associated migration patterns of cervical cancer cell lines. It is thus, conceivable that Oct4 is linked to cervical cancer metastasis, as reported for other cancer types, but further work is required to understand its involvement. These findings support, that not only do HPV(+) cervical cancers express higher levels of Oct4 compared to the HPV(-) cancers, but Oct4 is engaged in different molecular circuitry leading to inverse proliferation and migratory outputs compared to cancers which do not harbour HPV.

Oct4 has previously been linked to self-renewal in cancers [23]. We examined whether knockdown and overexpression of Oct4 can affect the ability of cancer cells to form tumorspheres and self-renew in tumorsphere assays, considered a proxy for the potential of cells to exhibit stem cell traits [23,35]. Our data show that in HPV(+) cervical cancer cells, Oct4 overexpression boosts their clonogenic and self-renewing ability, whereas knockdown of Oct4 has the opposite effect. Moreover, in all conditions tested, tumorspheres were always enriched for the expression of Oct4 as well as other stem cell factors supporting the notion that the cells tested represent *bona fide* cells with stem cell potential, and that high Oct4 expression is important for their self-renewal (S5 Fig). On the contrary, no strong difference was seen in HPV(-) cells. This reinforces the fact that proteins affecting stem-cell activity are not universal, and cannot necessarily translate across cell lines from similar cancer types.

In this manuscript, we provide evidence that the inverse Oct4-mediated phenotypes reported here in HPV(+) and HPV(-) cells are linked to the presence of the viral oncogenes without of course neglecting the genetic background of the cells. We show that introduction of the viral oncogenes into Oct4-knockdown HPV(-) cells, recapitulates to a large extent both the transcriptional as well as proliferation associated changes previously observed in HPV(+) cell lines in response to Oct4 knockdown. In addition, experiments conducted with non-transformed cells (HaCaT) further validate the Oct4-mediated phenotypes observed in HPV(+) cell lines. An *in vitro* interaction between Oct4 and HPV E7 was previously reported [25], however, recent proteomic work failed to identify Oct4 in the E7-host cell interactors, most possibly due to the low abundance of Oct4 in cells [36]. We provide the first piece of evidence that this interaction between E7 and Oct4 occurs in physiologically relevant cells and that the interaction maps to the CR3 region of the E7 protein. A point mutation at the position 67 of the HPV16 E7 protein changing the amino acid leucine to arginine (L67R mutant) completely abolishes this interaction. Nevertheless, it remains unclear whether the binding of Oct4 to the E7 protein in cancer cells is direct or indirect. In this manuscript we further show that the wildtype HPV E7 protein upregulates the mRNA expression of Oct4 in HPV(-) cells but the L67R mutant fails to do so (Fig 8D). We propose that the Oct4-E7 interaction in HPV(+) cells partially alters the Oct4-driven transcriptional output, which results in profoundly different outcomes on cell proliferation, migration, and potentially other phenotypes. In support of this, we provide evidence that the Oct4-mediated proliferation in HPV(+) cells is largely due to the interaction of Oct4 and E7. We show that transfection of the L67R E7 mutant in Oct4-expressing keratinocytes leads to increased proliferation, a phenotype in contrast to the attenuated proliferation recorded in Oct4 transduced keratinocytes expressing the wildtype E7. We thus conclude that the proliferative phenotypes linked to E7 are at least in part linked to Oct4 transcriptional targets and may further be linked to an interaction of E7 CR3 with Oct4 (Fig 9C and 9D, S8C Fig). We cannot exclude the possibility that additional domains of E7 are linked to the phenotype, or that transcription-independent effects [33] may be at play.

Additional information links Oct4 specifically to virally induced cancers. In HBV-related cancers, Oct4 is a marker of poor prognosis and has been shown to be upregulated via the IL-6 pathway [14]. Of interest, Oct4 has also previously been reported to interact with adenovirus E1A [25], raising the intriguing possibility that Oct4 represents a common target of oncogenic viruses which also impacts the viral lifecycles.

## Materials and methods

### Cell culture

Cervical carcinoma cell lines HeLa (HPV18), CaSki (HPV16) and C33A (HPV-negative) were purchased from ATCC and were maintained in 37°C and 5% CO_2_. HeLa and CaSki cells were preserved in DMEM and RPMI respectively, whereas C33A cells were cultured in MEM mixed with 1% L-glutamine. The 293T human epithelial kidney cells (ATCC) and HaCaT cell line (human immortalised keratinocytes) purchased from CLS (Cell Line Service) were cultured in DMEM. All cell culture media were supplemented with 1% penicillin-streptomycin (penstrep) and 10% fetal bovine serum (FBS). Primary mouse embryonic fibroblasts cultured in DMEM (MEFs) were isolated from E13.5 day C57/Bl6 embryos. For the reprogramming and cultivation of iPS cells, iPS medium was made by using DMEM supplemented with 15% knockout-serum, 1% penstrep, 1% b-mercaptoethanol, 1% non-essential amino acids (NEAA) and 0.1% leukemia inhibitory factor (LIF).

### Transfections—Lentiviral/retroviral transductions

C33A and HaCaT cells were plated at a density 1x10^5^ and 3x10^5^ respectively for transfection experiments. 24-hours post-seeding the cells were subjected to transfection with Fugene transfection reagent. For the transduction experiments, 293T cells were used at a density of 1x10^6^. Lentiviral or retroviral constructs were used as mentioned in S2 Table [42–50, 68–69]. The transfection of 293T cells was performed using Xtreme9 transfection reagent. 48 hours post-transfection the lentivirus/retrovirus was collected from 293T cells and applied to HaCaT, HeLa, CaSki and C33A with 1ug/ml Polybrene. Stable cell lines generated by retroviral transductions were selected with specific antibiotics as mentioned in S2 Table. For the generation of iPs, MEFs were plated and transduced with retroviruses expressing pMXs-Sox2 pMXs-Klf4 and pMXs-Oct4 or the HPV viral oncogenes. Transduced MEFs were grown in iPS medium for a couple of weeks until iPS colonies formed. Alkaline phosphatase staining was performed on fixed colonies using the Alkaline phosphatase detection kit according to the manufacturer’s instructions (Millipore SCR004). Positive foci with a diameter of ≥1mm were counted.

### Tissue Micro-Arrays (TMAs)

Tissue microarrays were obtained by Biomax.us (BC10025). TMAs were deparaffinised in xylene and rehydrated in a graded series of ethanol solutions. Antigen retrieval was done in a microwave using 10 mM citrate buffer. Blocking solution (5% horse serum) was incubated for 2h at room temperature. Primary antibody (Oct4 in a dilution 1:100) was incubated overnight at 4°oC. FITC-anti-rabbit secondary antibody was used at 1:300 for 45min at room temperature. All images were acquired using a Zeiss Axio Observer.A1 microscope. In tumors which were quantified as Oct4 positive >40–50% of the cells observed were positive.

### Reverse-Transcription-PCR and Real-Time PCR

RNA isolation was achieved by TRIZOL and the RNA extracts were treated with AMBION DNA-free to remove DNA impurities. The iSCRIPT cDNA synthesis kit was used to synthesise 300ng of cDNA whereas KAPA Taq PCR was used for reverse transcription PCR (RT-PCR). The real time PCR was achieved by KAPA SYBR FAST qPCR Master Mix (2X) kit from Kapa Biosystems according to the manufacturers’ guidelines and the primer sequences for performing both RT-PCR and Real-Time PCR are provided in S3 Table [51–67, 70–76]. For each gene, the average C(t) value was determined and was normalised to housekeeping (GAPDH) mRNA levels. Unpaired t-test (two-tailed) was used to calculate statistical significance.

### TCGA data extraction and RNA-seq analysis

RNA-seq data (read counts) were extracted from the Cancer Genome Atlas (TCGA) CESC dataset (containing 306 cervical cancer samples), using the Genomic Data Commons (GDC) Data Portal (https://portal.gdc.cancer.gov/). In addition, a pool of normal samples was extracted from the Genotype-Tissue Expression project (GTEx, 5 endo-cervical samples and 6 ecto-cervical samples) (http://www.gtexportal.org), the functional annotation of the human genome project (FANTOM5, one normal cervical sample) [37] (http://fantom.gsc.riken.jp/5/) and the Human Protein Atlas (HPA, one normal cervical sample) [38] (https://www.proteinatlas.org/), totalling 13 normal cervical samples. Read counts were normalized to transcripts per million (TPM) mapped reads, as previously reported [39]. Briefly, read counts were initially divided by the length of each gene in kilobases (reads per kilobase, RPK) and were then counted up and divided by 1,000,000 (“per million” scaling factor), producing the TPM values for each gene, in each tumor sample. A small offset (1) was added to avoid taking log of zero, as previously reported [39]. The log_2_(TPM + 1) scale was used to compare between cervical cancer and normal samples.The mRNA expression levels of *OCT4* (*POU5F1*) were evaluated using the limma R package with the cut-offs being log_2_FC = 1 and q-value = 0.01. The HPV status of each cervical cancer patient, along with other clinical data was retrieved from the Cancer Genome Atlas Research Network [40].

Regarding the analysis of the RNAseq data from the Oct4-knockdown HeLa and C33A cell lines, sequencing reads were filtered and mapped to the human reference genome (hg38) using HISAT2. On average ~93% of the reads were mapped to the reference genome. Following, htseq-count was used to count the aligned reads. The edgeR library was used to generate the counts-per-million (CPM) and filter them. The counts were then converted to DGEList object subjected to quality control. The library sizes were plotted as a barplot to see whether there were any major discrepancies between the samples. Box plots were used to check the distribution of the read counts on the log_2_ scale. Differential expression was performed using the limma R package with voom transformation. For statistical analysis the TMM was the method used to normalize library sizes. After voom transforming the data, differential expression (DE) analysis was applied between shOct4 and scrambled (control) cell lines, using the limma package. The limma topTable function was used to summarize the output in a table format. Significantly deregulated (DE) genes between shOct4 and scrambled cells were identified by selecting genes with a p<0.05 value and a fold change greater than 2 (FC>2). By default, the table was sorted by the B statistic, which is the log-odds of differential expression. The significantly up- and down-regulated genes in shOct4 against scrambled cell lines were depicted using Volcano plots. The top 20 DE genes were highlighted in blue. The data generated from the RNAseq experiment were validated by qRT-PCR. At least 8 highly regulated genes (randomly selected) from each comparison were initially confirmed. In addition to this, further genes from each comparison were used to establish the “Oct4 depletion signatures”. This resulted in a validation of a total of 23/23 genes for the C33A comparison, and 14/15 genes for HeLa comparison. One of the genes tested showed the anticipated trend but changes were not statistically significant. Gene expression changes confirmed for C33A (*ATP9A*, *KLF4*, *MYC*, *THBS1*, *ASNS*, *LEF1*, *ESAM*, *FYN*, *VGLL3*, *ALDH1A2*, *FN1*, *ITGB3*, *TERT*, *RERGL*, *FST*, *EIF4BP1*, *TGFA*, *TNFAIP3*, *LIMK1*, *RAC*, *PAK1*, *ACTB*, *CXCL8*. Gene expression changes confirmed for *HeLa* (*VGLL3*, *ALDH1A2*, *FN1*, *ITGB3*, *TERT*, *RERGL*, *FST*, *EIF4BP1*, *TGFA*, *TNFAIP3*, *LIMK1*, *RAC*, *PAK1*, *ACTB*, *CXCL8(ns))* (S8 Fig). The RNA sequencing analysis was performed once using three biological replicates. Unpaired t-test (two tailed) was used to calculate significance.

### Enrichment analysis

Gene Ontology enrichment analysis for the Biological Processes, Molecular Functions and Cellular Components of the top upregulated genes (log_2_FC>1) shOct4 HeLa and C33A cell lines, was performed using Enrichr [41].

### Western blot

Protein samples were extracted from immortalised keratinocytes and cervical cancer cell lines with RIPA cell lysis buffer (150mM NaCl, 5mM EDTA, 50mM Tris-HCL,1% TritonX-100, 0.1% SDS and 0.5% Sodium Deoxycholate) supplemented with Protease/Phosphatase inhibitors and their concentration was quantified and normalised by Bradford. For testing Oct4 expression in cancer cells, 60ug of protein extracts were measured and loaded onto a 12.5% SDS-PAGE gel for electrophoresis. For immortalised keratinocytes 100ug of protein extracts were used. The proteins were transferred on nitrocellulose membranes via a semi transfer apparatus and then membranes were blotted with 5% BSA for 60 minutes at room temperature and were incubated overnight at 4°C with the following primary antibodies ([1:1000 Oct4; Abcam, ab19857], [1:1000 GAPDH; Abcam, ab9484], [1:1000 Sox2 CST 3579], [1:250 HPV16E7; Santa Cruz, sc6981], [1:500 Lamin A/C; Santa Cruz, sc-6215] [1:500 GFP; Sicgen AB0020-200]. The blotted membranes were incubated with secondary antibodies conjugated with HRP (mouse anti-rabbit IgG HRP [sc-2357], mouse anti-goat IgG HRP [sc2354] and m-IgGk BP-HRP [sc-516102]) and were pictured using ECL reagents.

### Immunofluorescence

Cells were seeded on coverslips at a density of 1x10^5^. 24-hours post-seeding the cells were washed with sterile 1X PBS and then they were fixed with 4% paraformaldehyde. The cells were permeabilised with 0.2% Triton X-100 and blocked with a blocking buffer for 30 minutes. The Oct4 antibody (1:250) was diluted in blocking buffer and added to the cells overnight at 4°C. Then FITC anti-rabbit secondary antibodies were added at dilution 1:250 and incubated at room temperature for an hour. The blocked cells were washed with 1X PBS and visualised with fluorescent Zeiss microscope. For the staining of the cells’ nuclei the Vectashield antifade mounting medium with DAPI (Vector H-1200) was used.

### Generation of growth curves

Cervical cancer cell lines or HaCaT cells with stable expression of the Oct4 knockdown or Oct4 overexpression were seeded at a density 2x10^4^ or 2x10^5^ respectively in 6-well plates and counted manually using a hematocytometer every day for up to 10 days post-seeding (as indicated in respective graphs). Exclusion of dead cells using trypan blue was performed. Mann-Whitney U test (two-sided) was used to calculate statistical significance.

### Cell cycle analysis

For the cell cycle analysis of HeLa, CaSki and C33A cells, 1x10^6^ cells were harvested and fixed with ice-cold 70% ethanol and kept in 4°C for 2 hours. Then, fixed cells were collected, washed twice with ice-cold 1X PBS and treated with 0.2mg/ml DNase free-RNAse A. For staining the cells, 0.01mg/ml propidium iodide diluted in ice-cold 1X PBS was used for 30 minutes incubated in 37°C. The analysis was performed using Guava easyCYTE^TM^ Flow Cytometry (Millipore). Unpaired t-test (two-sided) was used to calculate statistical significance.

### Tumorsphere assay

For examining the clonogenic and self-renewing ability of cervical cancer cells, ultra-low attachment 6-well plates (Corning) were used for the seeding of 1x10^3^ cells/well. The cells were incubated for 2 weeks with DMEM/F12 combined with B27 supplement, 20 ng/ml basic fibroblast growth factor) and 20 ng/ml epidermal growth factor, in 37°C and 5% CO_2_. In order to examine the self-renewing abilities of the clones over 5 consecutive passages, the clones were collected, lysed with trypsin and then sieved through 40um cell strainers to create single-cells. Then single cells were re-plated as mentioned above. The clones were viewed and counted manually under the microscope. We considered all clones that were bigger than 70um in diameter. Unpaired t-test (two-sided) was used to calculate statistical significance.

### Wound healing assay

Serum starved cells at a 90% confluency were used for migration assays. To find the optimum FBS concentration to be used in the wound healing assay, HeLa, CaSki and C33A cells were treated with various serum concentrations. 48-hours post-treatment the number of cells in the serum-starved treatments was manually counted and compared with the number of cells in normal 10% FBS treatment. Consistent with literature we identified that the optimum serum concentration where cell numbers remain constant, is 0.5% (S6 Fig). Thereafter, the wound healing assay was performed. A p-20 tip was used to create a gap between the cell monolayer and the time that the gap was generated was termed as Time 0 (t = 0). The gap was photographed and measured using the AxioVision and Photoshop CS6 portable software at assorted time points until a full closure of the gap was noted. Mann-Whitney U test (two-sided) was used to calculate statistical significance.

### Co-immunoprecipitation

The immunoprecipitation experiments were conducted by immunoprecipitating endogenous Oct4 from HeLa Cells stably-expressing Flag-tagged HPV16E7, HPV18E7 and the control Flag-tagged GFP and stable C33A cells expressing the pLXSN-empty, pLXSN-HPV16E7 and pLXSN-HPV16E6E7 to verify protein-protein interactions in HeLa cells. The Cells were scraped off and lysed in ice-cold RIPA lysis buffer supplemented with protease/phosphatase inhibitors. Then, the mixture was centrifuged to extract the proteins. The lysate was then collected and some of it was separated to be used as whole cell lysate (WCL) control where whole cell extracts were quantified via Bradford assay. Then, the samples were subjected to a pre-clearing step where the lysates were incubated with the slurry (50ul slurry for 500ul lysate) for an hour under slow agitation. Then the mixture was centrifuged at 12000g for 20 seconds and the supernatant was collected and incubated overnight on a rotator at 4°C with Oct4 (1ug/ml), primary antibodies. The next day the slurry was added to the lysate-antibody mixture and kept for three hours on the rotator at 4°C. The beads and the antibodies were removed by boiling the samples at 95°C for 10 minutes and the precipitated lysate was collected and loaded onto a 12.5% SDS-PAGE electrophoresis gel. Then western blot for detecting protein-protein interactions (Oct4 with E7 or Lamin A/C or Sox2) was performed as indicated above.

### Microscopy

For the visualisation of cells the Zeiss Axio Observer.A1 microscope was used. For the quantification of the wound length in wound healing experiments the AxioVision and Photoshop CS6 software was used.

### Statistical analysis

Statistical analyses of the data were performed using the GraphPad Prism v.6.0 (La Jolla, CA). All the experiments were performed using at least three biological replicates and statistical significance was considered at p<0.05.

## Supporting information

S1 Fig Oct4 expression is up-regulated in HPV- related cancers mediated by the expression of the viral oncogenes. (A) Oct4 transcript levels are higher in HPV- positive Head and neck squamous cell cancer (HNSCC) compared to HPV- negative cases. (B) Oct4 levels are higher in HPV-16, HPV18 and HPV45 compared to HPV-negative cervical cancer. Oct4 mRNA levels differ between different HPV-subtypes. (C-D) HaCaT cells were transfected with E6 and E7 from various HPV types. The mRNA and protein levels of Oct4 were examined via qRT-PCR and Western blot respectively. (E) Semi-quantitative PCR reveals the successful transfection of HaCaT cells with the various HPV E6 and E7 constructs. Three independent experiments (mean±SEM) were used and statistical analysis was performed with Unpaired t-test (two-tailed) (ns = non-significant, *p<0.05, **p<0.01, ***p<0.001, ****p<0.0001).
(TIF)

S2 Fig Relative protein expression of Oct4 following lentiviral transductions. The values plotted on the graphs are the mean±SD and are taken from three independent replicates. (A) Oct4 protein levels in all three cervical cancer cells are significantly lowered in the stable knockdown condition compared to the scramble control. (B) Oct4 protein levels are elevated in the Oct4-overexpression condition compared to the controls. No statistical change was noted between the cherry & dox control compared to Dox control only. Unpaired t-test (two-tailed) was performed to calculate significance (ns = non-significant, *p<0.05, **p<0.01, ***p<0.001, ****p<0.0001).
(TIF)

S3 Fig Oct4 impacts the cell cycle of cervical cancer cells. (A) Cell cycle analysis was performed in HeLa, CaSki and C33A cells which express the Oct4 knockdown and Scramble control. Stable cervical cancer cells were fixed and stained with propidium iodide to identify the corresponding proportion of cells in the G1, S and G2/M phase of the cell cycle. Two-tailed Unpaired t-test was used and the data are taken form three independent replicates (ns = non-significant, *p<0.05, **p<0.01, ***p<0.001, ****p<0.0001).
(TIF)

S4 Fig Oct4 affects the migratory potential of HPV-positive and HPV-negative cells in an inverse way. (A) Optimisation of the amount of FBS to be added for the wound healing assays was made. 0.5% of FBS was found to keep the cell number steady after 48-hours of treatment. (B) Genes involved in the actin cytoskeleton pathway are deregulated upon stable Oct4 knockdown in HeLa and C33A cells reflecting the changes obtained in the wound healing experiments. Two-tailed Unpaired t-test was used and the data are taken form three independent experiments (ns = non-significant, *p<0.05, **p<0.01, ***p<0.001, ****p<0.0001).
(TIF)

S5 Fig Enrichment of stemness-related genes in tumorspheres formed from cervical cancer cells. **(A)** Phase-contrast images of the tumorpheres formed from adherent differentiated HeLa, CaSki and C33A cells (Scale bars, 200μm). (B) qRT-PCR was performed to examine the expression of stemness genes in the tumorsphere population compared to the monolayer of cervical cancer cells when Oct4 is overexpressed. Oct4, Sox2 and Klf4 are significantly enriched in the tumorspheres compared to the monolayer cells over the 4 generations tested. Statistical analysis of Unpaired t-test (two-tailed) was used (ns = non-significant, *p<0.05, **p<0.01, ***p<0.001, ****p<0.0001).
(TIF)

S6 Fig Bar graph visualization of the Gene Ontology (GO) enrichment results using Enrichr. The results show the top 10 enriched terms in (A) C33A and (B) HeLa and are sorted based on the combined score of the adjusted p-value and odds ratio. The most significantly enriched terms are noted in red colour of the bars (gray = non-significant terms, red = significant terms).
(TIF)

S7 Fig RNA-sequencing analysis reveals differentially expressed genes in Oct4-knockdown HPV-positive and HPV-negative cells. Volcano plots indicate a number of genes that were either upregulated or downregulated upon stable Oct4 knockdown in (A) C33A and (C) HeLa cells. (B&D) qRT-PCR was performed on a total of 8 genes (4 upregulated and 4 downregulated) to validate the data of the RNA-seq analysis. (E-G) qRT-PCR was performed to examine the percentage of the genes (15 genes in total) in Oct4-depleted C33A, C33A-E6E7 cells and Oct4-depleted C33A-E6E7 cells that match the HeLa Oct4-depletion signature. Two-tailed Unpaired t-test was used (ns = non-significant, *p<0.05, **p<0.01, ***p<0.001, ****p<0.0001).
(TIF)

S8 Fig Oct4 expression levels upon E7 expression in HaCaT cells. (A) Semi-quantitative PCR illustrates successful stable expression of pLXSN HPV16E6 and pLXSN HPV16E7 in Oct4-expressing HaCaT cells. (B) The validation of successful overexpression of Oct4 in HaCaT cells was made via a western blot. (C) Oct4-transduced keratinocytes were transfected with cmv-Neo Bam empty, cmv-16E7 and cmv-16E7 L67R mutant. The cells were harvested and examined for the protein levels of Oct4 via a western blot. (D) C33A cells transfected with cmv-16E7, cmv-16E7 L67R or GFP were used to immune-precipitate GFP. Interactions were visualised with Western blot. IgG was used as the negative control of the Immunoprecipitation experiment. GFP does not interact with Oct4 (E) Oct4 Knockdown and Scramble expressing C33A cells were transfected with cmv-16E7 and (F) cmv-16E7 L67R mutant. Gene expression was evaluated with qRT-PCR. Two-tailed Unpaired t-test was used (ns = non-significant, *p<0.05, **p<0.01, ***p<0.001, ****p<0.0001).
(TIF)

S1 Table HPV status in TCGA-CESC samples. (XLSX)

S2 Table List of plasmids used for transfection and transduction purposes. (TIF)

S3 Table Primer sets used for RT-PCR and qRT-PCR. (TIF)

S4 Table Differentially expressed genes in Oct4-knockdown C33A and HeLa cells. (XLSX)
