# Supplementary figures and images for: Human papillomavirus E7 binds Oct4 and regulates its activity in HPV-associated cervical cancers
Source: PLoS Pathog. 2020 Apr 16;16(4):e1008468. doi: 10.1371/journal.ppat.1008468 (PMC7228134; doi:10.1371/journal.ppat.1008468)

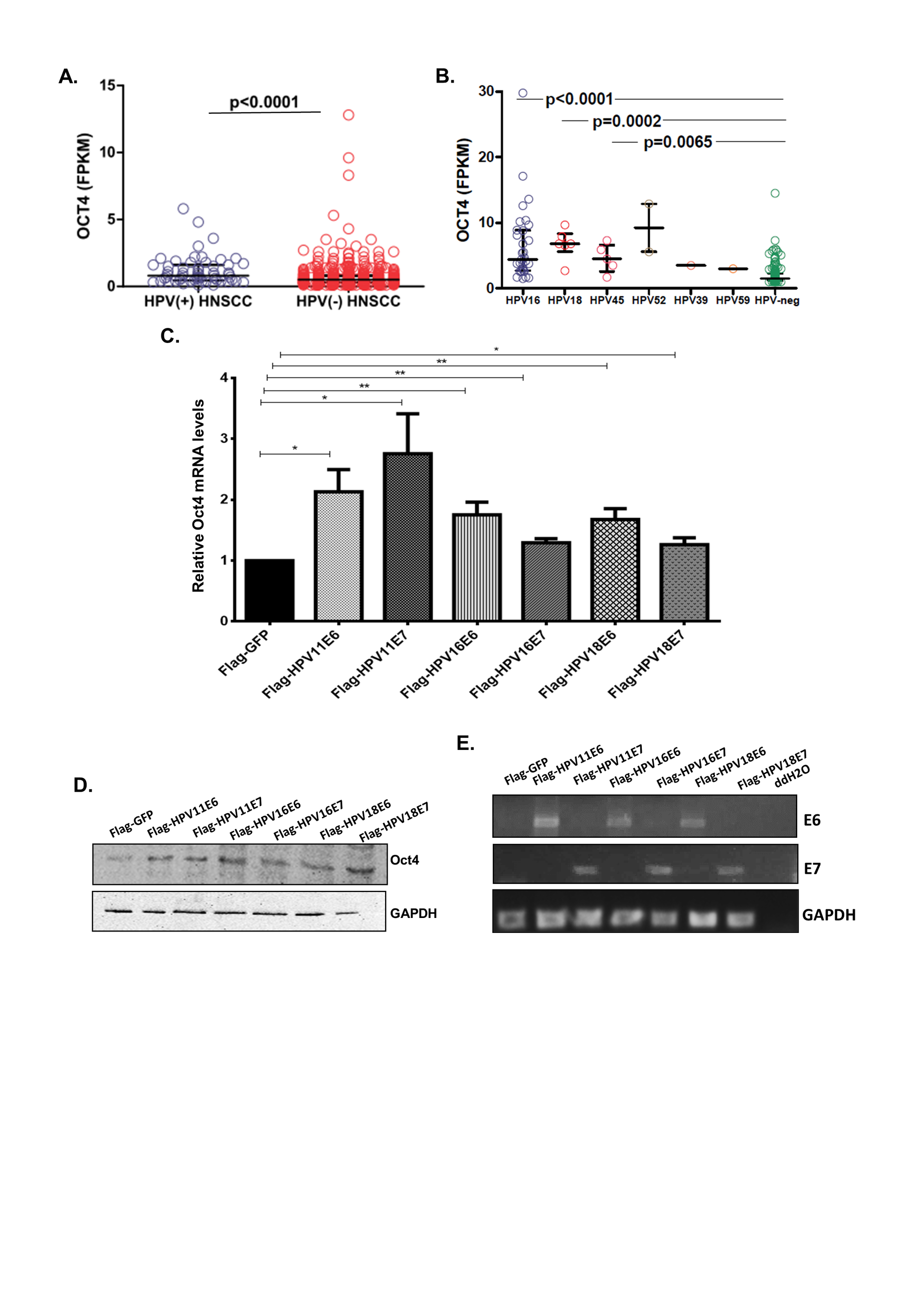

Supplement: S1 Fig — (A) Oct4 transcript levels are higher in HPV- positive Head and neck squamous cell cancer (HNSCC) compared to HPV- negative cases. (B) Oct4 levels are higher in HPV-16, HPV18 and HPV45 compared to HPV-negative cervical cancer. Oct4 mRNA levels differ between different HPV-subtypes. (C-D) HaCaT cells were transfected with E6 and E7 from various HPV types. The mRNA and protein levels of Oct4 were examined via qRT-PCR and Western blot respectively. (E) Semi-quantitative PCR reveals the successful transfection of HaCaT cells with the various HPV E6 and E7 constructs. Three independent experiments (mean±SEM) were used and statistical analysis was performed with Unpaired t-test (two-tailed) (ns = non-significant, *p<0.05, **p<0.01, ***p<0.001, ****p<0.0001). (TIF) [file ppat.1008468.s001.tif]

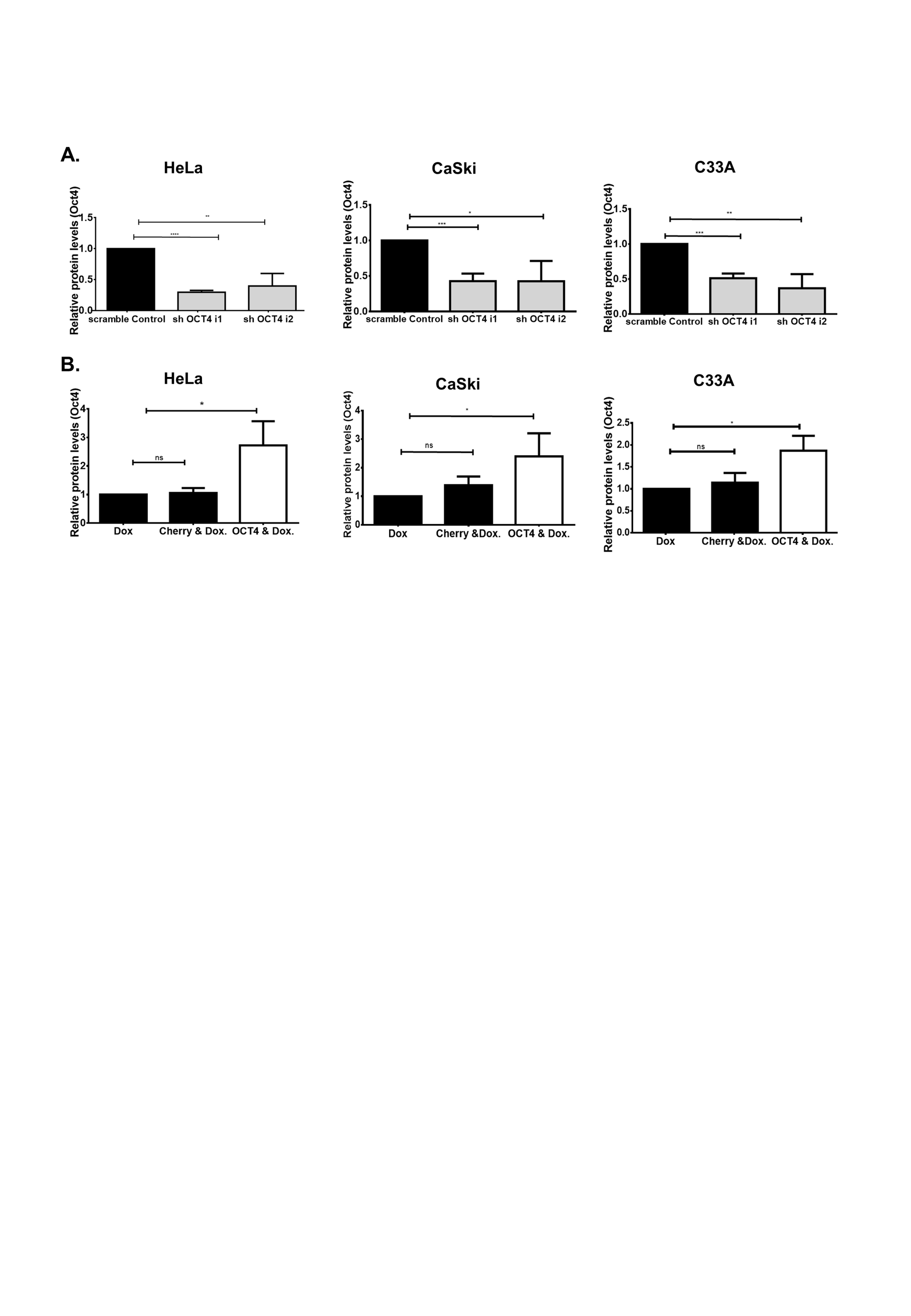

Supplement: S2 Fig — The values plotted on the graphs are the mean±SD and are taken from three independent replicates. (A) Oct4 protein levels in all three cervical cancer cells are significantly lowered in the stable knockdown condition compared to the scramble control. (B) Oct4 protein levels are elevated in the Oct4-overexpression condition compared to the controls. No statistical change was noted between the cherry & dox control compared to Dox control only. Unpaired t-test (two-tailed) was performed to calculate significance (ns = non-significant, *p<0.05, **p<0.01, ***p<0.001, ****p<0.0001). (TIF) [file ppat.1008468.s002.tif]

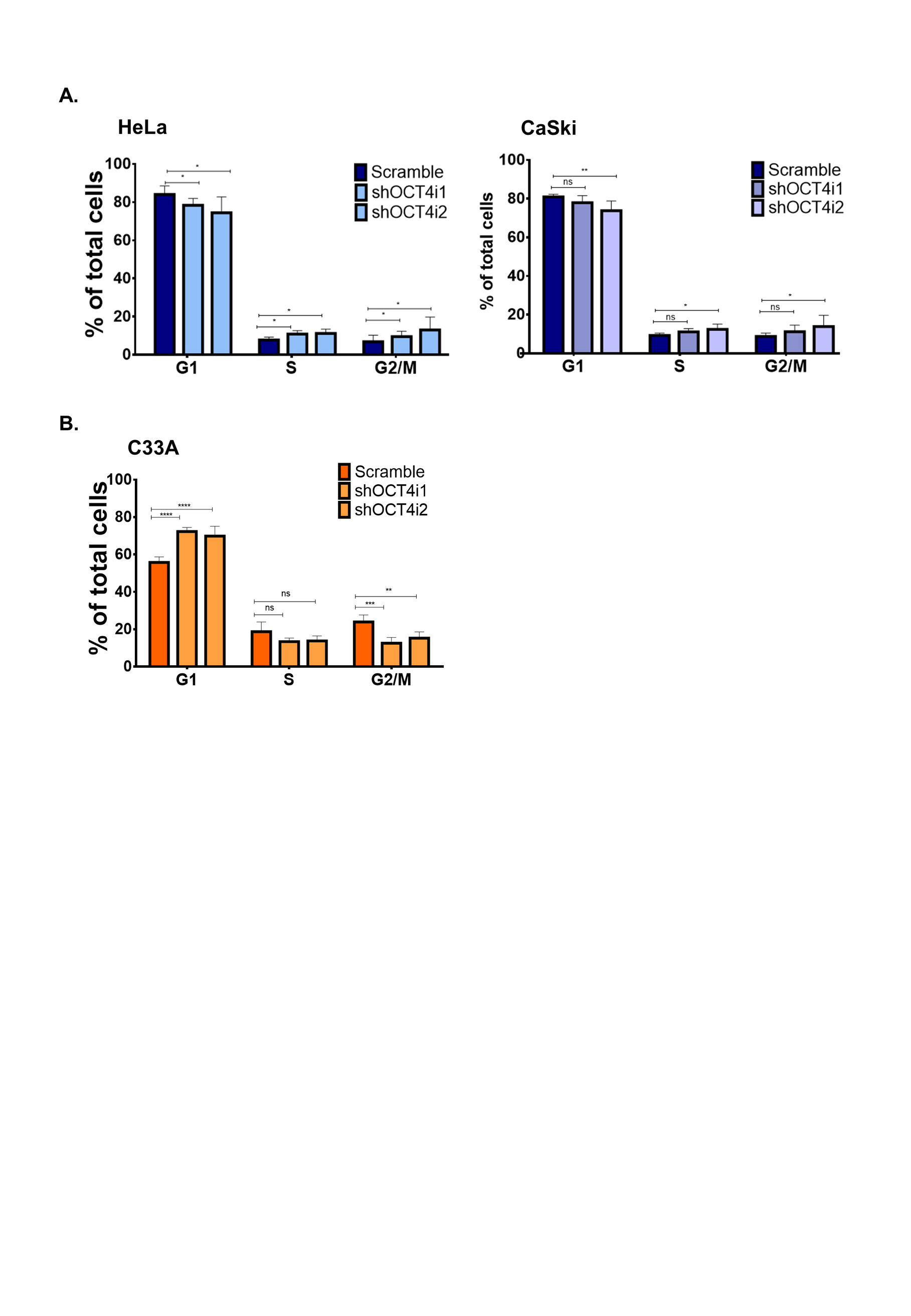

Supplement: S3 Fig — (A) Cell cycle analysis was performed in HeLa, CaSki and C33A cells which express the Oct4 knockdown and Scramble control. Stable cervical cancer cells were fixed and stained with propidium iodide to identify the corresponding proportion of cells in the G1, S and G2/M phase of the cell cycle. Two-tailed Unpaired t-test was used and the data are taken form three independent replicates (ns = non-significant, *p<0.05, **p<0.01, ***p<0.001, ****p<0.0001). (TIF) [file ppat.1008468.s003.tif]

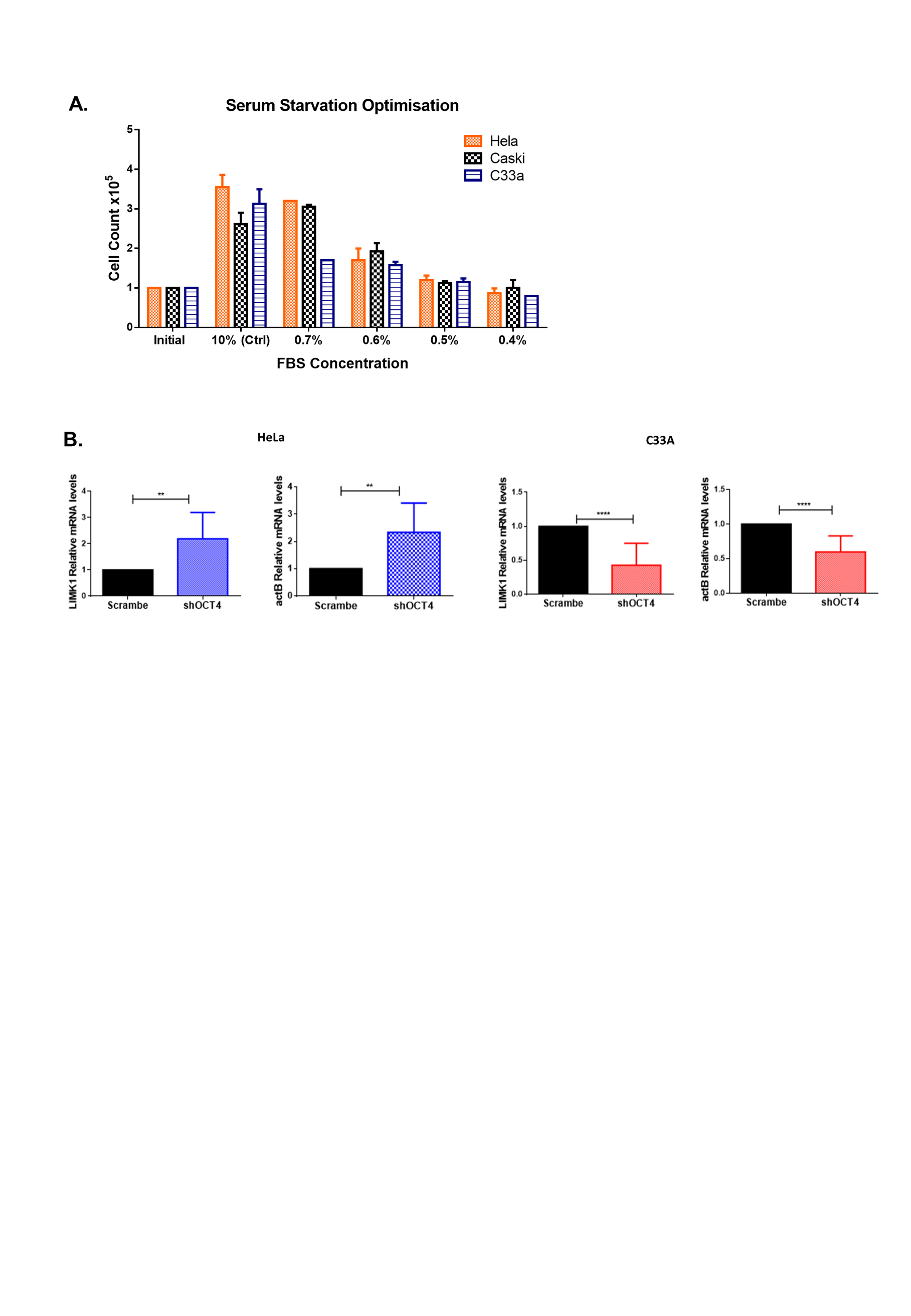

Supplement: S4 Fig — (A) Optimisation of the amount of FBS to be added for the wound healing assays was made. 0.5% of FBS was found to keep the cell number steady after 48-hours of treatment. (B) Genes involved in the actin cytoskeleton pathway are deregulated upon stable Oct4 knockdown in HeLa and C33A cells reflecting the changes obtained in the wound healing experiments. Two-tailed Unpaired t-test was used and the data are taken form three independent experiments (ns = non-significant, *p<0.05, **p<0.01, ***p<0.001, ****p<0.0001). (TIF) [file ppat.1008468.s004.tif]

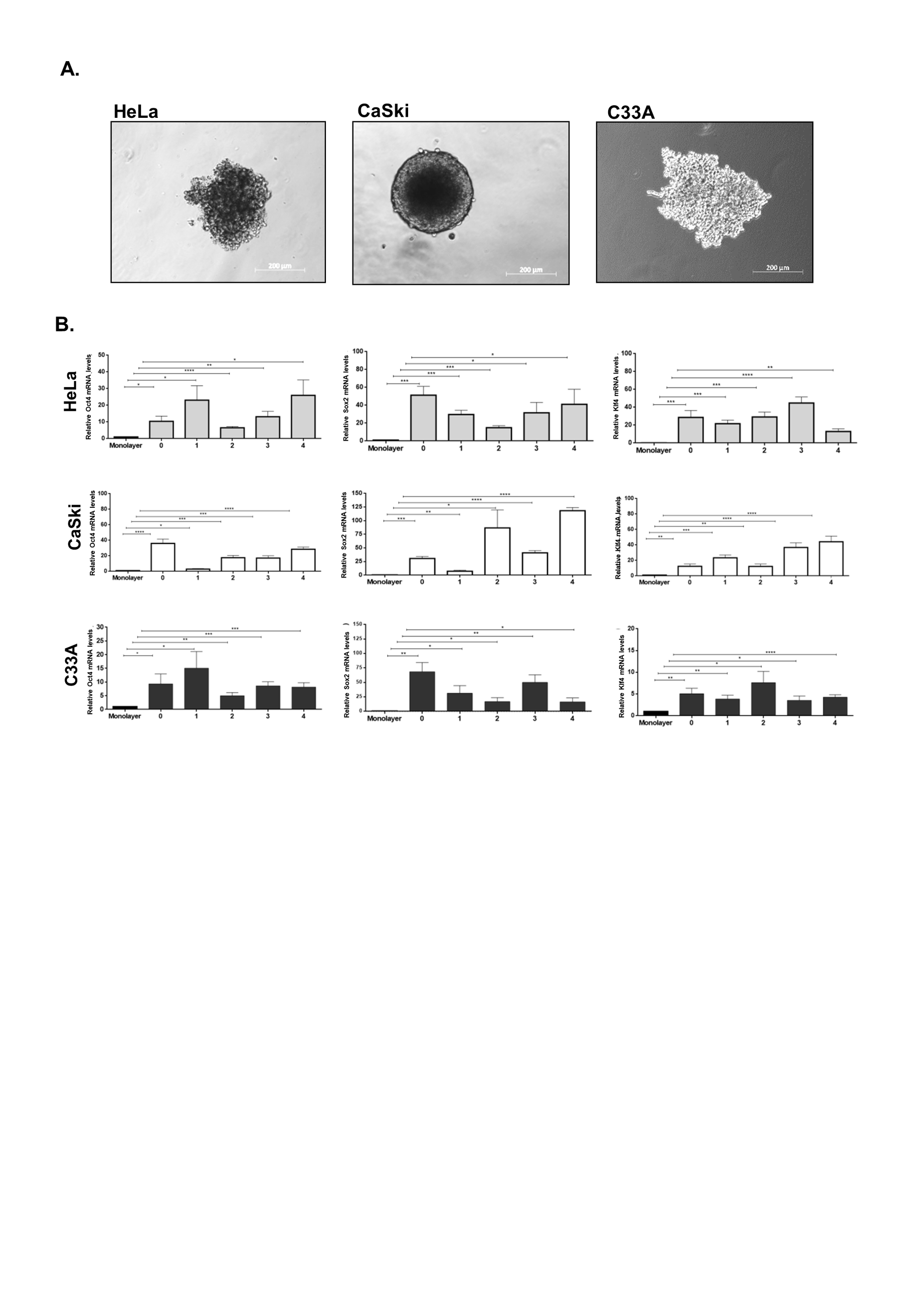

Supplement: S5 Fig — (A) Phase-contrast images of the tumorpheres formed from adherent differentiated HeLa, CaSki and C33A cells (Scale bars, 200μm). (B) qRT-PCR was performed to examine the expression of stemness genes in the tumorsphere population compared to the monolayer of cervical cancer cells when Oct4 is overexpressed. Oct4, Sox2 and Klf4 are significantly enriched in the tumorspheres compared to the monolayer cells over the 4 generations tested. Statistical analysis of Unpaired t-test (two-tailed) was used (ns = non-significant, *p<0.05, **p<0.01, ***p<0.001, ****p<0.0001). (TIF) [file ppat.1008468.s005.tif]

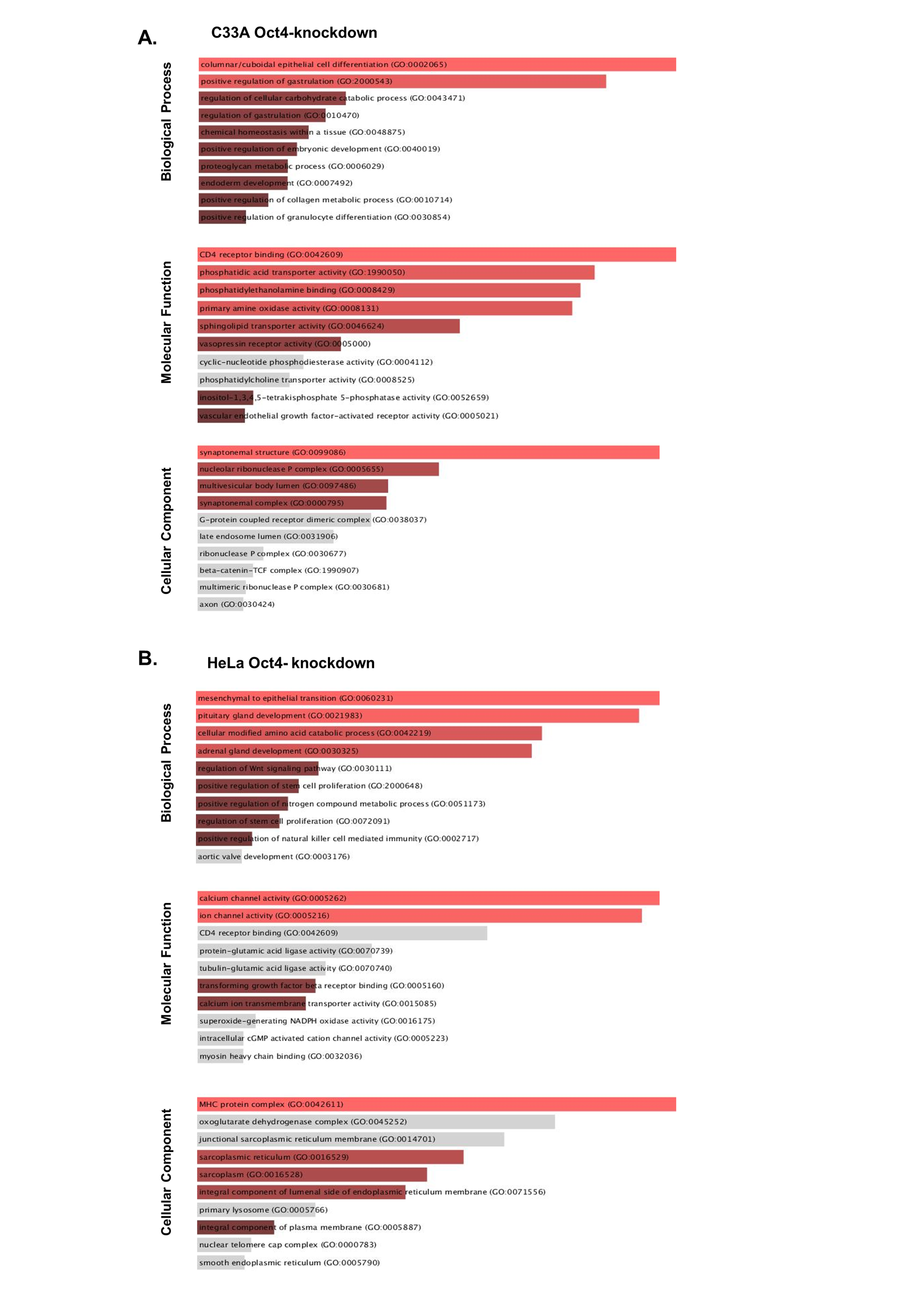

Supplement: S6 Fig — The results show the top 10 enriched terms in (A) C33A and (B) HeLa and are sorted based on the combined score of the adjusted p-value and odds ratio. The most significantly enriched terms are noted in red colour of the bars (gray = non-significant terms, red = significant terms). (TIF) [file ppat.1008468.s006.tif]

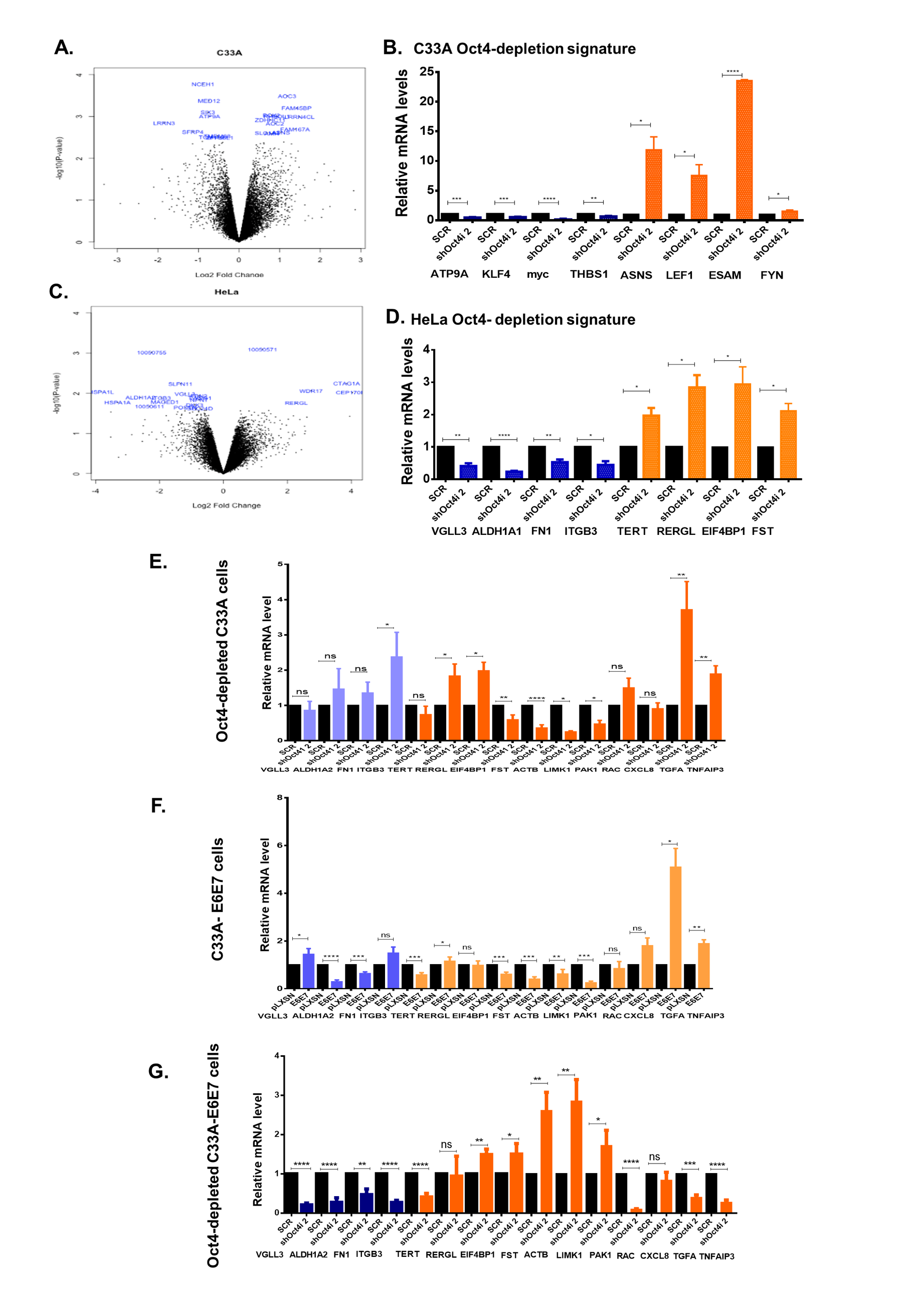

Supplement: S7 Fig — Volcano plots indicate a number of genes that were either upregulated or downregulated upon stable Oct4 knockdown in (A) C33A and (C) HeLa cells. (B&D) qRT-PCR was performed on a total of 8 genes (4 upregulated and 4 downregulated) to validate the data of the RNA-seq analysis. (E-G) qRT-PCR was performed to examine the percentage of the genes (15 genes in total) in Oct4-depleted C33A, C33A-E6E7 cells and Oct4-depleted C33A-E6E7 cells that match the HeLa Oct4-depletion signature. Two-tailed Unpaired t-test was used (ns = non-significant, *p<0.05, **p<0.01, ***p<0.001, ****p<0.0001). (TIF) [file ppat.1008468.s007.tif]

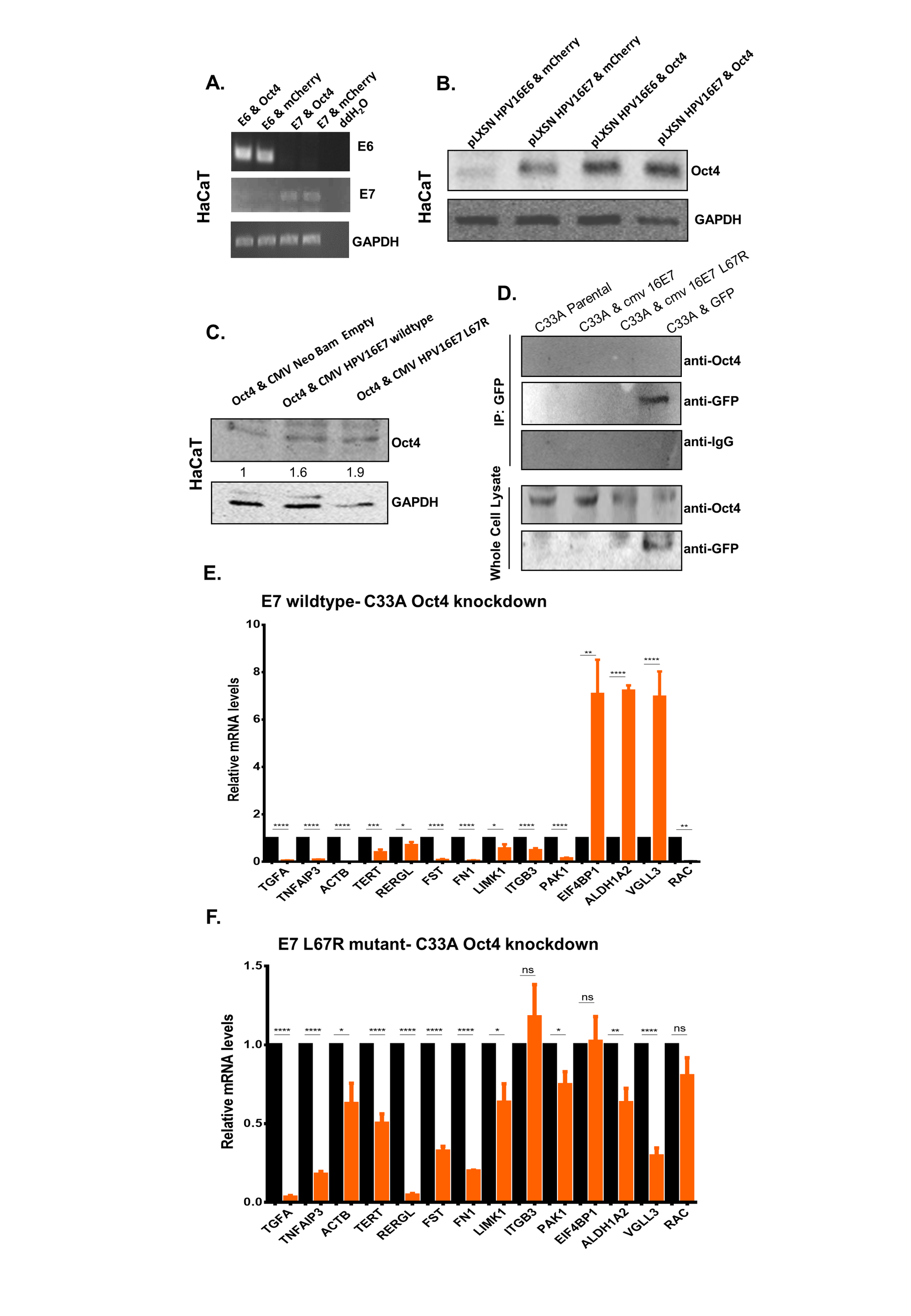

Supplement: S8 Fig — (A) Semi-quantitative PCR illustrates successful stable expression of pLXSN HPV16E6 and pLXSN HPV16E7 in Oct4-expressing HaCaT cells. (B) The validation of successful overexpression of Oct4 in HaCaT cells was made via a western blot. (C) Oct4-transduced keratinocytes were transfected with cmv-Neo Bam empty, cmv-16E7 and cmv-16E7 L67R mutant. The cells were harvested and examined for the protein levels of Oct4 via a western blot. (D) C33A cells transfected with cmv-16E7, cmv-16E7 L67R or GFP were used to immune-precipitate GFP. Interactions were visualised with Western blot. IgG was used as the negative control of the Immunoprecipitation experiment. GFP does not interact with Oct4 (E) Oct4 Knockdown and Scramble expressing C33A cells were transfected with cmv-16E7 and (F) cmv-16E7 L67R mutant. Gene expression was evaluated with qRT-PCR. Two-tailed Unpaired t-test was used (ns = non-significant, *p<0.05, **p<0.01, ***p<0.001, ****p<0.0001). (TIF) [file ppat.1008468.s008.tif]

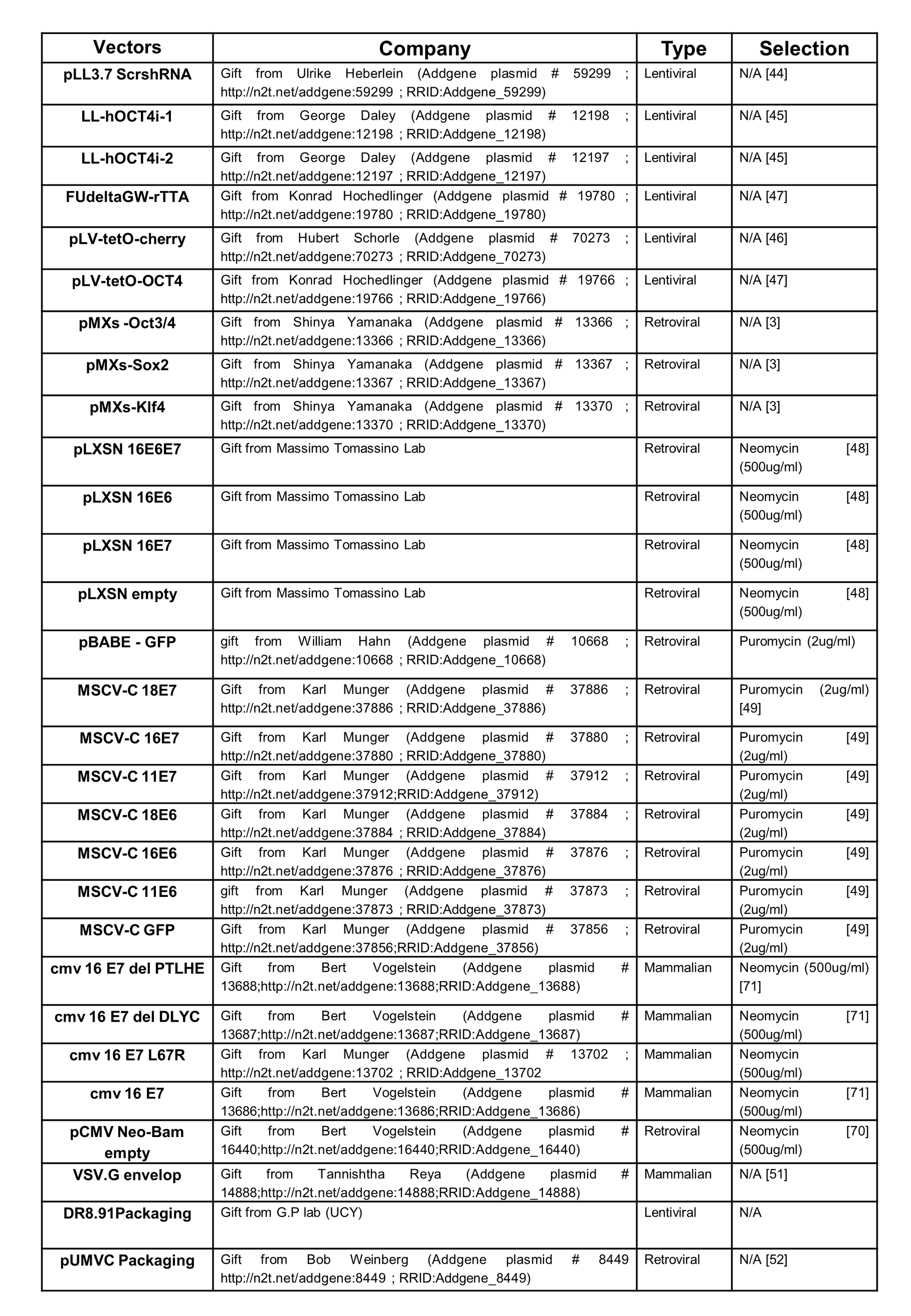

Supplement: S2 Table — (TIF) [file ppat.1008468.s010.tif]

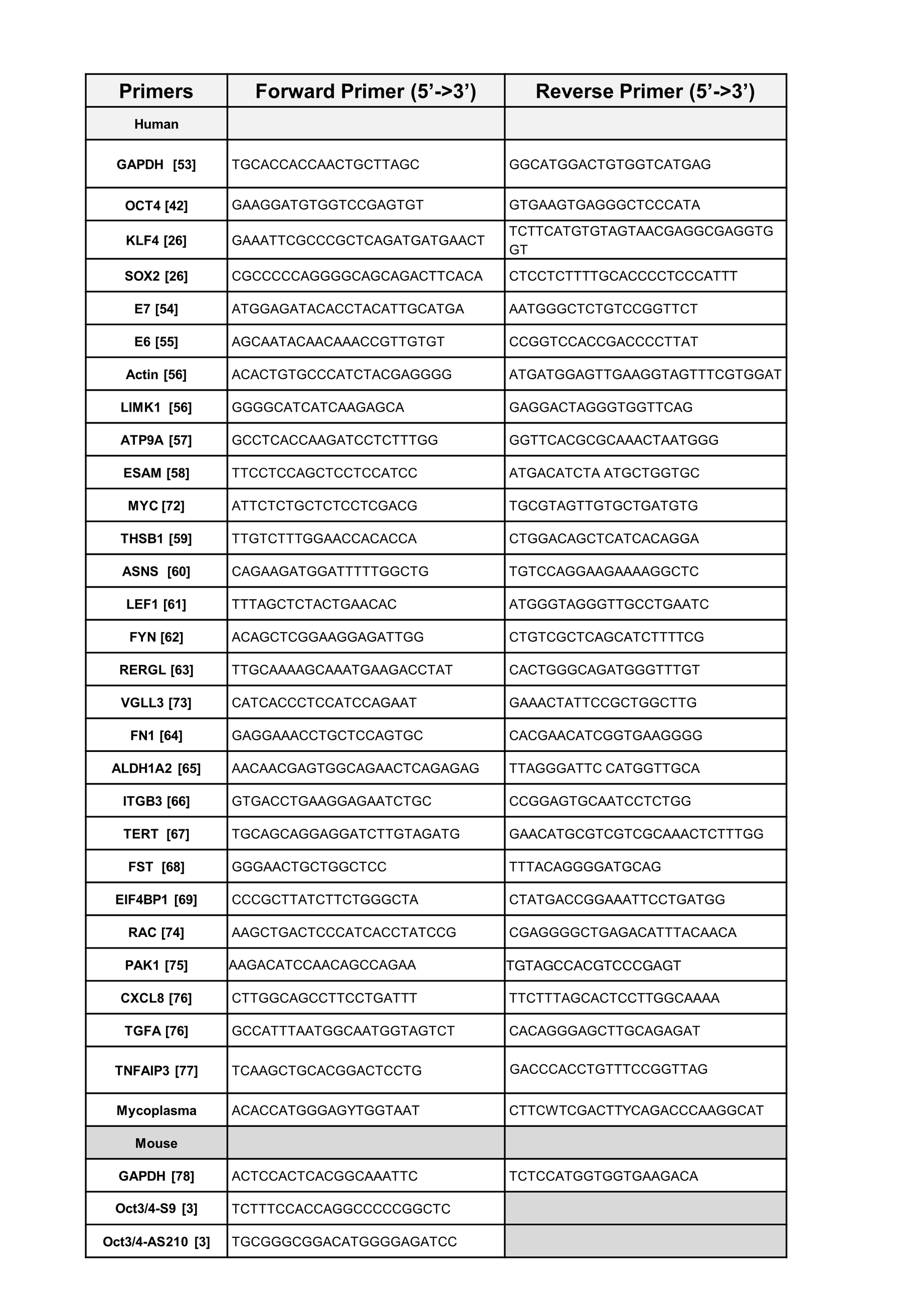

Supplement: S3 Table — (TIF) [file ppat.1008468.s011.tif]
